# Managing functional neurological disorder: treatment recommendations for health professionals in Australia

**DOI:** 10.1136/bmjno-2024-000970

**Published:** 2025-05-29

**Authors:** Alexander Lehn, Dharsha Petrie, David Palmer, Cindy Bradbury, Rianna Guest, Alana Schuurs, Jacinta Lewis, Rebecca Madden, Julia McLeod, Rodney Marsh, Christine Slade, Jessica Davis, Vince Cheah, Megan Broughton, Tjerk J Lagrand

**Affiliations:** 1 Department of Neurology, Princess Alexandra Hospital, Woolloongabba, Queensland, Australia; 2 Queensland University of Technology, Brisbane, Queensland, Australia; 3 STARS (Surgical Treatment and Rehabilitation Service), Brisbane, Queensland, Australia; 4 Department of Neurology, Sunshine Coast University Hospital, Sunshine Coast, Queensland, Australia; 5 The University of Queensland Queensland Brain Institute, Saint Lucia, Queensland, Australia; 6 Tools 4 Life Occupational Therapy, Brisbane, Queensland, Australia; 7 Department of Speech Pathology, Princess Alexandra Hospital, Woolloongabba, Queensland, Australia; 8 Gold Coast Hospital and Health Service, Southport, Queensland, Australia; 9 Logan Hospital, Loganholme, Queensland, Australia; 10 Queensland Health Metro South Pain Rehabilitation Centre, Brisbane, Queensland, Australia; 11 Griffith University The Hopkins Centre, Gold Coast, Queensland, Australia; 12 Tess Cramond Pain and Research Centre, Herston, Queensland, Australia; 13 Department of Consultation-Liaison Psychiatry, Royal Brisbane and Women’s Hospital, Herston, Queensland, Australia; 14 The University of Queensland Centre for Clinical Research, Herston, Queensland, Australia; 15 The University of Queensland Centre for Health Service Research, Woolloongabba, Queensland, Australia; 16 Neurology, Princess Alexandra Hospital, Woolloongabba, Queensland, Australia; 17 Brisbane Clinical Neuroscience Centre, Brisbane, Queensland, Australia; 18 Neurology, Alrijne Ziekenhuis Leiden, Leiden, The Netherlands; 19 Neurology, University Medical Centre Groningen, Groningen, The Netherlands

**Keywords:** FUNCTIONAL NEUROLOGICAL DISORDER, REHABILITATION

## Abstract

Functional Neurological Disorder (FND) can present significant management challenges due to its sometimes-complex presentation and the historical stigma attached to this diagnosis. Recent advances have improved understanding and management of FND, emphasising the benefit of a multidisciplinary approach to management. The prognosis of FND varies but evidence-based treatments offer the potential of remission to many people for whom FND might otherwise cause long-term disability, and meaningful symptomatic and functional improvement for many more. Despite this, limited and inequitable access to treatment means that many people with FND in Australia continue to experience treatable disability due to the condition.

1. Diagnosis: FND should be diagnosed based on positive signs rather than exclusion. This includes identifying inconsistencies and incongruencies in symptoms that differentiate them from other neurological conditions.
2. Communication: The diagnosis of FND should be communicated to patients promptly and clearly upon diagnosis of the condition. Information provided should include the name of the condition, the basis on which the diagnosis has been made, key principles that can aid self-management, and shared planning of next steps in treatment or accessing treatment.
3. Multidisciplinary Management: Across healthcare service models, treatment should involve a multidisciplinary team to address the multifaceted, and sometimes complex symptoms of FND.
4. Role of General Practitioners (GPs): GPs are integral in the long-term management of FND, providing continuity of care, patient support and education, and facilitating access to specialist services. An informed GP can provide the patient with confidence and agency to be pro-active in their symptoms.

Main Recommendations

1. Diagnosis: FND should be diagnosed based on positive signs rather than exclusion. This includes identifying inconsistencies and incongruencies in symptoms that differentiate them from other neurological conditions.
2. Communication: The diagnosis of FND should be communicated to patients promptly and clearly upon diagnosis of the condition. Information provided should include the name of the condition, the basis on which the diagnosis has been made, key principles that can aid self-management, and shared planning of next steps in treatment or accessing treatment.
3. Multidisciplinary Management: Across healthcare service models, treatment should involve a multidisciplinary team to address the multifaceted, and sometimes complex symptoms of FND.
4. Role of General Practitioners (GPs): GPs are integral in the long-term management of FND, providing continuity of care, patient support and education, and facilitating access to specialist services. An informed GP can provide the patient with confidence and agency to be pro-active in their symptoms.

Changes in Management as a result of the recommendations

The recommendations advocate for a shift from a pure psychiatric framework to a multidisciplinary and person-centred approach. Employing the biopsychosocial framework can enhance patient outcomes, including addressing protective and risk factors for Aboriginal and Torres Strait Islander people.

## Introduction

Functional neurological disorder (FND) is a brain processing disorder where neurological symptoms are caused by abnormal brain function rather than structural damage to the brain.^1^ It is among the most common diagnoses made in general neurology clinics and causes major disability.^2^ Despite this, guidance on managing FND remains limited.^3 4^ These recommendations aim to provide clinicians with foundational knowledge, emphasising the need for a multidisciplinary approach to understanding and treating FND in Australia.

## Methods

These consensus recommendations were developed by the Queensland Functional Neurological Disorder Special Interest Group (FND SIG). This group, established in 2019, includes 495 members from various healthcare specialties across Queensland, Australia. This article presents a condensed version of the more detailed recommendations, which are available as a supplementary document. The recommendations synthesise existing literature with expert opinion where the literature is inadequate. A process of repeated consultations with subspecialty groups within the FND SIG was set up, coordinated and overseen by a core guidelines committee. The body of scholarly literature for FND has increased in recent years, providing improved evidence for understanding the nature of FND as well as informing clinical practice. These guidelines use published evidence where possible; however, in areas where only scant research has been done, the recommendations reflect the working group’s experience and knowledge. This approach is based on guidelines for functional disorders published by the Danish College of General Practitioners (GPs) in 2013.^5^

### Patient and public involvement

The development of the recommendations involved extensive and invaluable consumer engagement and is endorsed by FND Australia Support Services. We are particularly grateful to Associate Professor Christine Slade, Ms Julie Wright and Dr Katherine Gill for their input on the development as people with lived experience with FND.

## Consensus recommendations

### Section 1: background on FND

#### Recommendations

- Understanding the prevalence of stigma against people with FND is important in relating to patients with the condition. A positive environment in which patients are supported and have the opportunity to ask questions and understand their condition is a prerequisite to engagement in treatment.
- Education about FND symptoms, diagnosis and management within a multidisciplinary context enables the best possible care for patients with a challenging and complex disorder.

#### Terminology

FND has been known by various names.^6^ The term ‘functional’ is preferred for its acceptability among patients, and positioning FND at the intersection of neurology and psychiatry.^7^ Patients with FND often experience scepticism and dismissal from healthcare providers, leading to a demoralising experience.^8^ Simply informing patients of their condition’s name can be profoundly empowering.^9^ Communicating this offers reassurance in the context of a challenging condition. It is important to recognise that FND is not voluntary and is not factitious disorder (feigning symptoms) or malingering.^10^ Such misconceptions unfortunately persist among some healthcare professionals and contribute to the stigma that people with FND frequently experience.^3 11^

#### Symptoms in FND

FND can cause a wide range of neurological symptoms, including dissociative attacks (also called functional seizures/dissociative seizures), gait problems, weakness, tremor, sensory disturbances, cognitive symptoms and swallow and communication difficulties. Patients often experience multiple symptoms simultaneously.^12^ Most symptoms tend to vary in intensity in proportion to symptom-focussed attention and to effort applied to overcoming them. Symptoms of FND frequently cause significant disability and often coexist with psychological comorbidities.^13^

The most common symptom groups are:

- **Dissociative attacks**: Involve altered movements, sensations and states of consciousness, which may resemble epileptic seizures.
- **Functional movement disorders**: Abnormal movements or postures affecting body parts, presenting as tremors, dystonia, myoclonus, tics, spasms, gait disorders or weakness.
- **Sensory disorders**: Often overlooked but can be significant symptoms impacting daily life, such as sensory illusions and visual symptoms.
- **Cognitive symptoms**: Includes both specific, effort-dependent cognitive symptoms which predominantly affect memory, known as functional cognitive disorder (FCD), plus the near-universal symptom of ‘brain-fog’.
- **Pain and fatigue:** Frequently occur alongside other symptoms of FND, although in the absence of other functional symptoms, they are not regarded as FND.

#### Prognosis

Untreated, the prognosis of FND is often poor, but it can be significantly improved with treatment.^14^ People with dissociative attacks achieve remission in 30%–50% of cases.^15 16^ In children, recovery rates are higher, with approximately 70% achieving seizure remission.^16^ Unfortunately, the lack of funding for treatment and skilled providers to deliver it means that the majority of people in Australia with FND are currently unable to access adequate evidence-based treatment.^17 18^

### Section 2: assessment and diagnosis

#### Recommendations

- The diagnosis of FND should rely on positive signs demonstrating fluctuation of symptoms with changes in symptom-focussed attention and/or effort to overcome the symptoms, incongruence with key features of non-functional diseases or both.
- FND is a clinical diagnosis based on history and examination. If positive features of FND are present on assessment, the diagnosis can be made with confidence and may not require further investigations.
- Generally, a diagnosis of FND should be made by a neurologist, ideally with specific interest in this area.

In making a formulation of any given FND presentation for the purposes of treatment, a broad person-centred approach based on a biopsychosocial model is essential to understand the complexity and individual variability of FND. Several interacting biological, psychological and social factors can influence vulnerabilities, triggers and perpetuating factors that contribute to FND as outlined in table 1.^19^

**Table 1:** The biopsychosocial model with potential factors that may contribute to FND (adapted from Nielsen *et al* 19)

Factors	Biological	Psychological	Social
Factors acting at all stages	Other neurological diseases History of previous functional symptoms	Emotional disorder Personality disorder	Socioeconomic deprivation Life events and difficulties
Predisposition (vulnerabilities)	Genetic factors affecting personality Biological vulnerabilities in the nervous system	Perception of childhood experience as adverse ­ Personality traits ­ Poor attachment/coping style	Childhood neglect or abuse Poor family functioning Symptom modelling of others
Precipitants (triggers)	Abnormal physiological event (drug side effect, hyperventilation, sleep deprivation) Physical injury/pain Anaesthesia/surgery	Perception of life event as negative/unexpected ­ Acute dissociative episodes/panic attacks	Social stressors/interpersonal conflict Bereavement
Maintaining factors	Plasticity in central nervous system’s motor and sensory pathways leading to habitual abnormal movements ­ Deconditioning ­ Neuroendocrine and immunological abnormalities similar to those seen in depression/anxiety Symptom-focussed attention caused by the symptom itself (vicious cycle)	Illness beliefs Perception of symptoms as being irreversible Not feeling believed Perception that movement will cause damage ­ Avoidance of symptoms ­ Fear of falling Health anxiety/concern about other potential diagnoses	Receipt of invalidity benefits Involvement in legal compensation processes Ongoing medical investigations and uncertainty Excessive reliance on wrong and unhelpful information which reinforce beliefs that symptoms are irreversible and purely physical in nature

FND, functional neurological disorder.

#### Assessment

Clinicians should base the diagnosis of FND on positive signs demonstrating fluctuation of symptoms with changes in symptom-focussed attention or effort to overcome the symptoms, incongruence with key features of non-functional diseases, or both. Examples include:

- Physical signs demonstrating inconsistency between impaired voluntary movement and intact automatic movement, for example, Hoover’s sign in functional lower limb weakness or entrainment in functional tremor.
- Attacks with an appearance typical for a dissociative attack which have no EEG correlation.
- Memory difficulties which are much worse on direct testing of memory than when (automatically) recalling details in conversation.

#### Diagnostic approach

Neurologists, particularly those with relevant expertise, are ideally positioned to diagnose FND, although other specialists can also accurately diagnose the disorder. Misdiagnosis is rare when handled by skilled professionals, but it should be noted that FND can frequently coexist with non-functional disorders, and structural conditions often trigger FND.^20–22^ Clinicians should remain alert to features that would suggest a second, admixed, diagnosis (eg, epilepsy and dissociative attacks).^21 23^

### Section 3: treatment and management

Most patients with FND benefit from treatment. For symptoms that are mild and of recent onset, a short period of observation to allow for the spontaneous remission of symptoms after initial diagnosis and explanation is sometimes appropriate. Patients who do not improve over this period, or who have severe or long-standing symptoms, usually require structured interventions.^24^ Treatment should be initiated by the diagnosing specialist and will ideally be supported by GPs and allied health professionals.

#### GP role

##### Recommendations

- GPs should be informed about the diagnosis of FND and equipped with strategies to support ongoing patient management.
- The GP–patient relationship is pivotal as a means of providing care in the longer term. GPs should have access to specialist advice and be given the opportunity for continuing professional development in the management of FND.

Long-term management often requires sustained involvement from GPs who play a vital role in patient care. Access to necessary support for both the GP and the patient with FND is often hindered by limited availability of public services and the financial burden of private care.^25^ The patient journey can be isolating, often requiring patients to become their own healthcare advocate, stay informed about new research and treatments and educate clinicians with limited FND knowledge.^26 27^ A supportive and proactive GP can significantly improve the patient journey, and other specialists should not underestimate the value of such therapeutic relationships. GPs should be included in multidisciplinary teams and provided with opportunities for ongoing professional development.

Finding a knowledgeable and sympathetic GP can sometimes be challenging due to a myriad of factors. Patients living in regional and rural areas may have additional challenges due to difficulties in attracting and retaining GPs.^28^ In these cases, telehealth consultations with GPs who are more experienced in FND may provide a viable solution.

#### Neurologist role

##### Recommendations

- The neurological assessment can be seen as the first step in FND treatment, not just a prelude to diagnosis.
- Effective explanation of a diagnosis of FND can alter key beliefs and is an opportunity to teach some preliminary self-management strategies that patients can implement immediately.
- Neurologists have a role in triaging to different types of evidence-based treatment.

The neurological assessment serves both diagnostic and therapeutic purposes. The primary goal is to establish the diagnosis, which should be followed by a clear explanation of FND as this can significantly improve patient outcomes.^9^ While not dwelling on the possibility of other causes, it should directly address any specific patient concerns that the symptoms might be due to another cause (eg, a brain tumour or multiple sclerosis). Addressing these concerns early and assertively usually prevents the need for further investigations as a means of reassuring the patient.

Neurologists can recommend multidisciplinary approaches to help patients manage their symptoms before seeing other health professionals, for example, basic physiotherapy or psychology techniques. These interventions can activate the process of educating and empowering patients to take ownership of their treatment plan and hopefully see improved symptom management.

#### Physiotherapist role

##### Recommendations

- Physiotherapy management should include facilitating normal movement, retraining normal movement, addressing secondary changes and education (including role of physiotherapy, activity pacing and long-term self-management of symptoms).

Physiotherapy can be effective for managing functional movement disorders in both inpatient and outpatient settings.^29 30^ The subjective assessment should aim to understand the patient’s symptoms, including biopsychosocial triggers and symptom patterns over the day; gauge the patient’s acceptance and understanding of their FND diagnosis; and serve as an opportunity to jointly set management goals.^31^ The focus of the objective assessment should be on activity performance and functional ability, such as mobility, rather than on impairment measures. This approach may reveal positive signs that aid consolidating the diagnosis of FND. In selecting objective measures, the effect of attention on symptoms should be borne in mind, and measures of function rather than specific movement should be preferred.

Movement retraining as treatment should be goal-oriented and focused on facilitating self-management.^30^ Distraction techniques are recommended in movement retraining for FND. These techniques involve engaging patients in competing movements, weight-bearing activities or purposeful limb use. Distraction should be used to provide an alternative sensorimotor experience rather than attempting to deceive the patient. Management also includes providing education about FND, energy conservation, pain management, caregiver and family education and developing a relapse prevention plan.^19^

#### Occupational therapist role

##### Recommendations

- Occupational therapists can assist patients with FND manage difficulties in day-to-day function and improve participation.

Occupational therapists play a crucial role in the multidisciplinary and biopsychosocial care of individuals with FND based on their expertise in both physical and mental health and their focus on function and participation.^32^

Occupational therapy for FND emphasises re-engagement in daily activities with interventions including motor retraining and desensitisation, energy conservation, graded activity resumption, sensory modulation, vocational rehabilitation, psychosocial intervention and education to support self-management. Engaging patients and their families in problem solving and developing strategies to enhance function is essential.^33^

#### Speech pathologist role

##### Recommendations

- Speech pathologists are uniquely qualified to assess speech, voice, language, cognitive-communication and swallowing disorders. This allows for a major contribution to the diagnosis and effective treatment of these functional symptoms in patients with FND.

Speech pathologists have a critical role in diagnosing and managing FND when functional speech, voice, language, cognitive communication, swallowing and cough are significant symptoms. They are adept at identifying the functional nature of these symptoms and explaining the diagnosis to the patient.^34^ As with other symptoms of FND, functional communication, swallowing and cough disorders may exhibit inconsistencies over time, across different tasks and with distraction. While speech pathologists can sometimes manage these symptoms independently, involving a multidisciplinary team is recommended if progress is limited or if additional symptoms are present, ensuring comprehensive care and improved patient outcomes.^35^

#### Psychologist role

##### Recommendations

- Psychologists provide direct treatment of FND symptoms by targeting thoughts, feelings, physiology and behaviour that may be maintaining symptoms. Cognitive–behavioural therapy (CBT) has the largest evidence base for treating FND.
- Clinical psychologists can diagnose and treat coexisting psychological conditions (ie, anxiety, PTSD) that may drive FND symptoms.
- Clinical neuropsychologists are experts in the diagnosis and management of cognitive disorders (including FCD).

Research on effective psychological interventions for FND is growing.^36^ Psychological therapy is recommended to address factors contributing to FND and persistence of symptoms. Common mechanisms identified include dissociation, differences in emotional processing and sense of agency, difficulties in emotion regulation and altered interoceptive awareness. Psychologists can also identify underlying psychiatric disorders and comorbidities to guide clinical formulation and treatment.

While research on CBT for FND symptom reduction/remission is mixed, the impact on secondary outcomes such as improved quality of life and psychological function is positive.^37^ Evidence for expressive psychotherapies is limited, highlighting the need for more prospective trials. Psychologists can identify underlying psychiatric disorders and comorbidities to guide clinical formulation and treatment.

When cognitive concerns are a predominant presenting feature, a referral to a clinical neuropsychologist familiar with FCD is recommended to improve diagnostic clarity and guide clinical management.

#### Psychiatrist role

##### Recommendations

- Consider a referral to a psychiatrist for diagnosis and treatment of psychiatric comorbidities in patients with FND.

The primary responsibility of psychiatrists in FND treatment is to identify and treat comorbid psychiatric diagnoses with evidence-based psychotherapy and/or pharmacotherapy. Evidence for specific pharmacological treatment for FND is limited, and psychotropic medication should be prescribed cautiously.^1^

Psychiatrists may be involved in the diagnosis of FND (ie, determining that the symptoms are not better explained by another psychiatric condition such as a dissociative disorder).^38^ Psychiatrists have an important role in the ongoing treatment of some people with FND through psychoeducation, managing comorbid psychiatric conditions and collaborating with the patient’s family and healthcare team.^39^

Traditionally, FND has been thought to be a downstream consequence of trauma, although this connection is not universally applicable.^40 41^ Where present, trauma-informed psychotherapy by an appropriately trained psychiatrist or psychologist may be helpful for some people with FND. Psychiatrists may also provide psychotherapy as an element of continuing patient care.

The involvement of different types of psychiatrists can vary depending on local service models and clinician expertise. General psychiatrists typically work across a broad range of psychiatric conditions and may contribute to FND management by diagnosing comorbid disorders (eg, anxiety, depression, Post-traumatic stress disorder (PTSD)) and initiating pharmacological or psychotherapeutic treatment where appropriate. Liaison psychiatrists, who are based in hospital settings, are often consulted in the context of diagnostic uncertainty or when functional symptoms present in acute care. They may also support the initial engagement of patients with the FND diagnosis and facilitate referrals to longer-term care. Neuropsychiatrists, who specialise at the interface of neurology and psychiatry, are ideally placed to work with patients with complex FND presentations, particularly where there is diagnostic uncertainty. However, access to neuropsychiatry services in Australia remains limited, particularly outside of metropolitan areas.

#### Social worker role

##### Recommendations

- Social workers can provide practical skills and support to promote better health outcomes for people with FND and their supporting networks.

Social workers play a multifaceted role in the management of FND, including advocacy, psychosocial assessment and care planning. They assist patients in understanding their diagnosis, developing self-care strategies and accessing community resources. Their interventions are designed to validate patients’ experiences, enhance emotional regulation and support adjustment to the disorder.

#### Nursing role

##### Recommendations

- Nurses can play a vital role in the care of patients with FND by providing appropriate support and monitoring, both in inpatient and community settings.

Nurses play a crucial role in the care of inpatients with FND, being able to implement multidisciplinary care plans and addressing concerns in the absence of other healthcare team members across a 24-hour period. As a result of the consistent and relatively intense patient interaction, nurses’ attitudes towards FND have a significant impact on patients and their families. They can assist patients in identifying triggers, monitoring progress, supporting biopsychosocial needs and enhancing health literacy for patients and their families. For dissociative attacks, nurses collaborate with doctors to ensure there are emergency call criteria and management plans that are well documented and clearly communicated from shift to shift.

Outpatient and community nurses can help patients with FND navigate complex healthcare systems. They can facilitate access to services, serve as a point of contact for troubleshooting outpatient therapies and provide support in home environments. Community nurses may also assist patients in accessing resources such as Centrelink, National Disability Insurance Scheme (NDIS) and GP management plans to gain access to further therapy.

#### Rehabilitation physician role

##### Recommendations

- Rehabilitation physicians and their teams can provide specialised care for patients with FND focused on maximising a patient’s independence and quality of life.

Evidence from inpatient and outpatient settings indicates that rehabilitation often provides at least short-term functional benefits, with many patients experiencing longer-term improvements.^42^ Rehabilitation physicians focus on diagnosing and assessing functional impairments to maximise people’s independence and improve and maintain quality of life. In the context of FND, rehabilitation physicians can play an important role, especially where there are barriers to accessing neurologists. They often diagnose FND, provide education, validate patient experiences, manage coexisting medical concerns and develop multidisciplinary treatment plans. They collaborate with patients and multidisciplinary teams to set realistic and flexible rehabilitation goals to guide treatment in the context of fluctuating symptoms.^43^

#### Troubleshooting common multidisciplinary challenges

Not all patients with FND benefit from allied health support to optimise their function. Effective therapy is usually contingent on a clear diagnosis, patient receptiveness to the diagnosis, and willingness to participate in therapy.^1^

Assistive devices are generally not recommended during the acute phase of rehabilitation due to the risk of reinforcing symptoms, but these considerations should be balanced against patient safety, as injuries from falls do occur in people with FND.^19^ When necessary, such devices should be considered short-term solutions, with plans to progress from their use with treatment.

Diagnosing and managing FND typically requires multidisciplinary collaboration.^44^ All health professionals involved can observe target symptoms, report positive signs and engage the multidisciplinary team in assessment and management. In acute settings, this includes paramedics, triage nurses and emergency department (ED) staff. FND patients may come with a history of clinical disbelief. ED staff have a significant role to play, not only in assessing physical symptoms, but in assuring the patient that they believe what they are saying about their illness. This positive approach can be life-changing for the patient, giving them the best chance of early improvement.^45^

In community settings, collaboration with GPs, physiotherapists and other allied health professionals is crucial for comprehensive care. Even without a definitive diagnosis, applying FND treatment approaches can be beneficial for people where FND is a leading differential diagnosis for their symptoms.

Effective communication and patient education on symptom management enhance treatment acceptance and engagement.^46^ Multidisciplinary team meetings are helpful for addressing arising concerns and care planning. Collaboration with experienced therapists and professional societies offers additional support and training. Discharge from multidisciplinary services should focus on self-management, with clear information on resources, goals and follow-up plans.^44^ Ensuring patients are equipped with self-management skills is essential, as relapses are common.

#### Managing FND with limited resources in regional and remote communities

Managing FND in Australian regional and remote communities is challenging due to limited resources, reduced familiarity with FND due to the breadth of generalist practice and difficulty accessing training for clinicians.^17^ Outcomes for people with FND in these settings have not been well studied, although outcomes for most patients with chronic illnesses are worse in rural areas.^47^ Despite these challenges, effective management is possible with accurate diagnosis and clear explanations, which can be provided by a local doctor with specialist support. Milder cases may benefit from targeted therapy by local therapists or a multidisciplinary team with specialist support. Improved communication and support from more experienced centres, along with telehealth services, can mitigate the vast geographical barriers and enhance access to treatments (ie, psychology, physiotherapy and psychiatry).^48 49^ Increased funding and training can help develop resources and upskill clinicians in these regions.^2^

#### FND in Aboriginal and Torres Strait Islander people

A few considerations for FND in Aboriginal and Torres Strait Islander people are important. For example, protective and risk factors must be considered in the broadest possible sense, including social determinants of health, local community and culture and the impact of current and historical systems on trust. Also, expectations need to be managed accordingly in this group of patients. FND can be a highly conceptual and challenging concept. Management often involves reframing symptoms to create a healing rather than damaging outlook. Given the many barriers that exist between Indigenous and non-Indigenous people, good communication is a cornerstone of management.

#### FND and the NDIS

Accessing support for people with FND from the NDIS presents challenges due to the condition’s variable nature and uncertain prognosis, particularly surrounding access criteria where a person’s impairment needs to be deemed permanent.^27^ This scheme is unique to Australia. A second concern is that the change from rehabilitative treatment to disability support can contribute to the perpetuation of symptoms. Furthermore, NDIS decision-makers lack medical expertise and have limited knowledge of FND. Successful funding applications typically require specialist reviews and comprehensive multidisciplinary documentation.

While FND is not an acknowledged condition by the NDIS, funding may be accessible for chronic, refractory cases where there is significant psychosocial disability. The DSM-5-TR (Diagnostic And Statistical Manual Of Mental Disorders, Fifth Edition, Text Revision) criteria classify the condition as persistent if symptoms are occurring for 6 months or more.^50^ Collaboration within the multidisciplinary team is crucial, with documentation detailing that evidence-based treatment has finished and has not led to a substantial improvement in the person’s functional capacity. Documenting subjective and objective measures over multiple time points can be used to demonstrate a long-term and permanent pattern of disability impacting on the person’s functional capacity, ability to work, study or participate in social life.

### Section 4: resources

#### Troubleshooting common multidisciplinary challenges

##### For healthcare professionals

Managing FNDs: Consensus recommendations for the management of FND that have been developed by the Queensland Functional Disorder Special Interest Group (FND SIG) and are endorsed by FND Australia Support Services Inc: https://fndaustralia.com.au/resources/FND-treatment-recommnedations-FINAL-20-May-2024.pdf

FND Standard of Care: This document was published by the Department of Rehabilitation Services at The Brigham and Women’s Hospital. It outlines recommended care practices and clinical guidelines for professionals treating FND: https://www.brighamandwomens.org/assets/BWH/patients-and-families/rehabilitation-services/pdfs/functional-neurological-disorder-standard-of-care.pdf

##### For healthcare professionals and patients

Neurosymptoms (https://neurosymptoms.org/en/): This site is a trusted resource for patients and health professionals. It provides extensive evidence-based information and guidance on FND symptoms and management with many downloadable factsheets and a new online FND Formulation Tool for patients.

Head injury symptoms (https://headinjurysymptoms.org): This webpage has been developed by the same team as neurosymptoms and clinically relevant information as well as practical tips on mild head injury, concussion and mild traumatic brain injury.

FND Australia (https://fndaustralia.com.au): The website of the Australian FND network offers resources for both health professionals and patients. It includes patient information, educational videos, online learning courses, a healthcare professional directory, other resources for professionals and a patient workbook to support management.

##### For patients

National Disability Advocacy Program: Through the National Disability Advocacy Program, patients receive guidance on accessing services like the NDIS, Centrelink and other government supports. More information can be found here: https://www.dss.gov.au/disability-advocacy/national-disability-advocacy-program.

FND Australia Support Services (https://fndaus.org.au): Offers peer support, educational programmes and resources for FND patients and carers. Key services include a low-fee counselling and OT programme for eligible patients, the ‘Kokoro Mollitia’ education and well-being programme, and FND Awareness Day events. Accessible nationwide via Zoom, the organisation provides continuous support to improve patient care and well-being.

FND Hope (https://fndhope.org): An international support network offering education and advocacy for people affected by FND.

Functional Seizure Management Plan: Created by FND Australia Support Services Inc and developed with input from FND experts. This plan guides carers and emergency responders in supporting patients with functional seizures. A PDF version of the plan can be downloaded here: https://fndaus.org.au/functional-seizure-management-plan/.

##### Apps for patients

FND Aus App (https://fndaus.org.au/fnd-app/): A free app with information, symptom tracking and a functional symptom management plan to help patients manage acute symptoms like functional seizures. It also allows for sharing progress with healthcare teams.

Neurosymptoms FND Guide (https://neurosymptoms.org/en/): An app version of the *Neurosymptoms* website, offering a patient-friendly guide to FND symptoms and treatment.

Calm (www.calm.com), Headspace (www.headspace.com) and Smiling Mind (www.smilingmind.com.au) Apps: These apps provide tools for mindfulness, meditation, sleep and relaxation, helping patients manage stress and enhance overall well-being.

## Conclusions

The management of FND requires a comprehensive, multidisciplinary approach tailored to the individual needs of patients. Although in Australia significant progress has been made in understanding and treating the condition, barriers such as limited access to specialist care, particularly in regional areas, continue to hamper the delivery of adequate care for most patients. Enhancing professional education, increasing public awareness and improving access to multidisciplinary services for all Australians are essential steps to ensure that all individuals with FND receive timely and effective treatment. With continued research and collaboration, it is likely the management for patients with FND will be improved, offering hope for better outcomes and quality of life.

10.1136/bmjno-2024-000970.supp1 Supplementary data
