# Supplementary material for: Managing functional neurological disorder: treatment recommendations for health professionals in Australia
Source: BMJ Neurol Open. 2025 May 29;7(1):e000970. doi: 10.1136/bmjno-2024-000970 (PMC12184360; doi:10.1136/bmjno-2024-000970)
Supplement: Supplementary data [file bmjno-7-1-s001.pdf]

MANAGING FUNCTIONAL NEUROLOGICAL DISORDERS

A State-wide Recommendation for an FND Clinical Pathway

Queensland, Australia

May 2024

This consensus recommendation has been developed by the Queensland Functional Disorder Special Interest Group (FND SIG) and is endorsed by FND Australia Support Services Inc. The authors would like to thank the many FND SIG members for their time and valuable contributions. We particularly thank Dr. Christine Slade, Ms Julie Wright and Dr. Katherine Gill (who all have lived experience with FND) for their extensive and invaluable contribution to this body of work.

Version History

Version	Revision date	Summary of Changes
1.0	1 March 2024	Initial publication

Please cite as:

Managing Functional Neurological Disorders - A State-wide Recommendation for an FND Clinical Pathway. The Queensland Functional Neurological Disorder Special Interest Group, May 2024.

Table of Contents

Table of Contents 3
The Queensland Functional Neurological Disorder Special Interest Group 7
Preface 8
Levels of evidence and strength of recommendations 9
Section 1. Background to Functional Neurological Disorder (FND) 10
Introduction 10
Symptoms in FND 11
Historical perspectives of, and terminology for FND 12
Does feigning or malingering occur in FND presentations? 13
Prognosis of FND 13
Section 2: Assessment and Diagnosis 14
Assessment 15
Barriers to an effective assessment in FND 15
What are the most important aspects of history taking? 15
Can talking about FND or testing for it make the symptoms worse? 16
Assessing a patient for positive symptoms of FND 16
Should you perform additional investigations in patients with FND? 17
Diagnosis 17
Are there diagnostic criteria for a Functional Neurological Disorder? 17
How is the diagnosis of FND made? 18
Who can make the diagnosis of a Functional Neurological Disorder? 18
Differential diagnosis and comorbidity 18
How likely is a diagnosis of FND to be correct? 18
Does a diagnosis of FND rule out other underlying disorders? 19
Section 3. Treatment and management 20
The Role of the General Practitioner 20
The role of the neurologist 21
What is the role of neurological assessment in the treatment of FND? 21
How can relapses of FND be prevented and pre-emptively treated? 21
Are there any materials that a neurologist or other healthcare professional can refer to? 22
The role of the physiotherapist 22
What is the role of the physiotherapist in the treatment of FND? 22
What are the physiotherapy assessment options for various functional symptoms? 22
Subjective Interview 22

Objective assessment 23
Suggestions for isolated symptoms 23
Outcome measures 24
What are the physiotherapy treatment options for functional motor symptoms? 24
The role of the occupational therapist 25
What is the role of the occupational therapist in the treatment of FND? 25
What are the occupational therapy assessment options for various functional symptoms? 25
What is the current evidence for occupational therapy (OT) intervention? 25
Assessment and goal setting 25
OT Treatment 26
Motor symptoms 26
Sensory symptoms 26
Cognition 26
The role of the speech pathologist 27
What is the role of speech pathologists in the diagnosis and management of FND? 27
What are the speech pathology assessment options for various functional symptoms? 27
General speech pathology assessment features 27
Voice 27
Fluency 28
Articulation 28
Language and cognitive-communication 28
Swallowing 28
What are the speech pathology treatment options for various functional symptoms? 29
Voice 29
Fluency 29
Articulation 29
Language and cognitive-communication 29
Swallowing 29
The role of the psychologist 30
What is the role of the psychologist in the treatment of FND? 30
What is psychology’s role in diagnosis? 30
How is CBT used in the context of FND? 31
The role of the psychiatrist 31
What is the role of the psychiatrist in the treatment of FND? 32
When is pharmacological treatment indicated in FND? 32
The role of the social worker 33
Is there a role for a social worker in FND? 33

What are the different roles for a social worker in FND? 33
Advocacy 34
Psychosocial assessment and care planning 34
Therapeutic/support intervention & adjustment/validation 34
The role of the nurse 35
What's the role of a nurse with an inpatient with FND? 35
What is the role of a nurse (navigator) in the community for patients with FND? 35
The role of the rehabilitation physician 36
What are the key aspects of the role and scope of a rehabilitation physician? 36
What is the role of a rehabilitation physician in the diagnosis and management of FND? 36
Multidisciplinary management of FND 37
When is it appropriate to refer to allied health? 37
When is it appropriate to trial and use assistive devices? 37
I have been referred a patient without a definitive diagnosis of FND / I suspect my patient has FND. 38
General approach 38
Inpatient setting 38
Community setting 38
How will my team handle complexity? 39
General advice regarding complex cases 39
Are there services available for patients with severe and intractable functional symptoms? 39
When is the right time to discharge a patient from multidisciplinary services? 39
Return to work/school 40
Access to disability support and navigating the NDIS 40
Eligibility for NDIS 40
FND in elderly patients 41
Can elderly people have FND and how common are FND symptoms in the elderly patient? 41
What are the most common presentations of FND in the elderly? 42
What are the red flags or clues to look for in considering FND in the elderly? 42
How to diagnose and treat FND in the elderly patient? 42
FND in Aboriginal and Torres Strait Islander people 42
How do you include the patient's background in the cross-cultural treatment situation, particularly considering Aboriginal and Torres Strait Islander people? 42
What are helpful considerations when communicating with Aboriginal and Torres Strait Islander people? 43
Health care systems in FND 43

Is a doctor needed as part of the FND treatment team? 43
What should be done if a patient with FND presents acutely to hospital? 44
What are the models of care for therapy of FND and how can they be accessed in Queensland? 44
What is available to help with the hospital to community transition on discharge? 45
Current Queensland Health specialised FND programs 45
Horizons Program – Gold Coast University Hospital 45
FND Education Program – Cairns Base Hospital 45
FND Clinic – Sunshine Coast University Hospital 45
Private FND services in Queensland 46
How to determine when and which sub-acute rehabilitation service is required? 46
How can I manage FND with limited resources in regional and remote communities? 47
FND co-existing with persistent pain 48
Is there a clinical overlap between persistent pain and FND? 48
When should I refer patients to neurology/rehabilitation services vs a persistent pain clinic? 48
Where do I start when treating patients with FND with co-existing persistent pain? 48
References 50
Resources 54
Online Resources 54
For health care professionals 54
For patients 55
Patient Support Groups 55
FND Australia Support Services Inc – fndaus.org.au 55
FND Hope – fndhope.org 55
Useful Apps for patients 55
FND Aus App 55
Neurosymptoms FND Guide 55
Calm app and headspace app 56
Smiling Mind app 56
Professional Memberships 56

The Queensland Functional Neurological Disorder Special Interest Group

The Queensland Functional Neurological Disorder Special Interest Group (FND SIG) has been established in 2019. Membership in the special interest group is available to Queensland Health, Department of Education or non-government organisation clinicians and other identified key stakeholders in Queensland. Currently the FND SIG has 495 members from all Queensland health services across a wide variety of healthcare specialties.

The special interest group was set up to connect health professionals in Queensland who work with patients with Functional Neurological Disorders to:

- Provide support and advice to Queensland clinicians managing patients with FND
- Share knowledge, expertise and resources regarding managing FND
- Develop FND educational resources for therapists and patients
- Streamline and develop pathways for FND services for patients across the state
- Build and sustain a network of clinicians across Queensland working in FND management
- Collaborate and coordinate state-wide evaluation strategies, education and research opportunities in FND

To achieve these goals the FND SIG provides regular online and in-person professional development for its members. It facilitates and supports research projects in the field of Functional Neurological Disorder through mentoring and research grants. The SIG also hosts an annual FND symposium for members and other interested clinicians for clinical and research updates as well as networking opportunities. The SIG also publishes written and online educational resources for use in clinical practice.

The Queensland Functional Neurological Disorder Special Interest Group is an official partner of the international Functional Neurological Disorder Society.

To contact the FND SIG please email the secretary on FND.SIG@health.qld.gov.au.

Preface

Although Functional Neurological Disorders (FND) can cause severe disability and patients with FND are frequently seen by health professionals, there is currently little guidance in managing this condition. Most health professionals have received little to no training in the diagnosis or treatment of patients with FND. The diagnosis of FND can carry significant stigma and unfortunately many health professionals still have an attitude that patients with FND are feigning their symptoms. This situation can be exacerbated by doctors who are uncomfortable with their neurological examination skills of patients with functional symptoms and then being unsure how to communicate a diagnosis of FND. There is an increasing body of evidence regarding diagnosis and effective treatment. In many patients the diagnosis of FND can be made accurately based on hard neurological signs observed on examination with a single sound explanation having a significant therapeutic effect for patients, making acceptance easier and allowing low-cost targeted interventions to help many patients. Marked and sustained improvements have been seen in patients with functional movement disorders after multidisciplinary inpatient rehabilitation as well as outpatient-based therapy[1,2]. Psychological therapy in the form of cognitive behavioural therapy for patients with dissociative attacks has been shown to lead to improved quality of life and better global functioning[3].

It is encouraging to see the increasing number of health professionals who are interested in Functional Neurological Disorders despite often finding these patients difficult to help. A commonly raised concern is the lack of guidance about how to approach common issues in this area. Guidelines about diagnosis and management of FND have been published in other countries in the past[4], but for this publication we wanted to create something that is pragmatic and addresses specific issues we face in Queensland, Australia. These guidelines are intended to support a broad audience of health professionals involved in the diagnosis and management of patients with FNDs in different demographics and contexts, from generalists in rural settings to specialist multidisciplinary services in tertiary centres. The hope is also that as our FND Special Interest Group grows and we build up further expertise, we will be able to continue developing these guidelines, so they remain relevant for the future.

While we cover basic knowledge about current concepts of FND and underlying mechanisms leading to FND in these guidelines, the main focus is everyday clinical practice. These guidelines are not exhaustive, and many areas are not covered (or are only covered superficially). We trust these guidelines can provide a foundation for health professionals to build upon when managing patients with FND and serve as a scaffold for future versions.

Levels of evidence and strength of recommendations

'Guidelines translate best evidence into best practice'[5]. The purpose of grading the strength of the evidence and recommendations is to make it transparent for the user the basis for the recommendations in the guidelines. Table 1 provides a schema for interpreting the level of evidence for each recommendation. The recommendations' strength is graded from A (greatest validity) to D (least validity). In the guideline, the recommendations' strength is indicated to the left in the box. When evaluating the validity of the underlying knowledge, one must bear in mind that not all knowledge can be verified in randomised trials. This approach is based on the excellent guidelines for functional disorders that were published by the Danish College of General Practitioners in 2013[6].

The body of scholarly literature for FND has increased in recent years as research interest rose, providing significant evidence for improved understanding of the nature of FND as well as informing clinical practice. Yet, while these guidelines are supported by evidence as much as possible, there are areas where only scant research has been done and so the recommendations reflect the working group's experience and knowledge (see table below).

Recommendation	Level of Evidence	
**A**	1A	Systematic review (with homogeneity) of RCTs
	1B	Individual RCT (with narrow confidence intervals)
	1C	All or none study
**B**	2A	Systematic review (with homogeneity) of cohort studies
	2B	Cohort studies
	2C	Database studies
	3A	Systematic review (with homogeneity) of case-control studies
	3B	Case-control studies
**C**	4	Uncontrolled studies, case reports
**D**	5	Expert opinion without explicit critical appraisal or based on pathophysiology, laboratory research or rule of thumb
✅	5	Recommended by the writing group as 'good clinical practice'

Lehn A, *et al. BMJ Neurol Open* 2025; 7:e000970. doi: 10.1136/bmjno-2024-000970

Section 1. Background to Functional Neurological Disorder (FND)

Recommendations	
✅	Accepting that FND is a disorder in which patients' symptoms are real provides: Consistency in the clinical approach to patients who may have already experienced skepticism from medical practitioners. A positive helping environment in which patients are supported and have opportunity to accept their condition and possibly improve.
✅	Being educated about FND systems, diagnosis and management within a multidisciplinary context enables the best possible care for patients with a challenging and complex disorder.

This section includes foundational information to assist clinicians to have basis treatment knowledge about FND. Clinician interaction with FND patients varies in intensity levels depending on disciplinary and contextual factors. However, having a broad transdisciplinary clinical understanding and acceptance of what is FND, the range of symptoms that can be experienced and appreciating the turbulent medical mis(understanding) of FND, makes an enormous positive outcome for the patient's interaction with the medical system.

Introduction

Functional Neurological Disorder (FND) is a brain processing disorder where neurological symptoms are caused by abnormal brain function rather than structural damage to the brain[7]. Clinically, its hallmark is inconsistency; this is seen both between symptoms and signs, and in the fluctuation of signs and symptoms over time. Attention can have a striking effect on both symptoms and signs, causing worsening with attention and improvement with distraction[8].

No single process has been identified as being sufficient to explain the onset of FND. Research suggests that the quality of parent/child interaction, disorganised attachment, and neglect early in life can lead to development of dissociative disorders irrespective of trauma[9,10]. It is also important to point out that trauma or difficult childhood experiences are only risk factors: Many patients with FND do not report previous traumatic experiences and most people who have had traumatic past experiences do not develop FND later in life[11].

FND is one of the most common diagnoses in general neurology clinic, and the second most common reason for referral to a neurologist after migraine/headache[12]. The lower estimates of prevalence in the community are around 50 people per 100 000 population[13].

In a well-designed consecutive series of 3781 outpatients of neurology clinics, 5.4% had a primary diagnosis of FND, and 30% had symptoms that were described as only somewhat or not at all explained by disease[14].

In an Australian study of an outpatient neurology clinic, 15% of all the new presentations received a primary diagnosis of FND[15].

A recent study done at an emergency department in Queensland showed that more than 25% of the patients with presumed epileptic seizures, actually had dissociative attacks[16].

Lehn A, *et al. BMJ Neurol Open* 2025; 7:e000970. doi: 10.1136/bmjno-2024-000970

Women are more frequently affected by FND and are estimated to be 60 to 75% of the patient population, although specific presentations such as functional myoclonus or functional Parkinsonism appear to have similar or greater frequency in men[13].

Symptoms in FND

Almost any neurological symptom can be caused by the condition, but common symptoms include dissociative attacks (aka psychogenic non-epileptic seizures (PNES) or functional seizures), gait problems, weakness, tremor, and sensory disturbance[17].

Patients tend to have multiple symptoms simultaneously. In an international patient survey (n=1048) by Butler et al. (2021) less that 1 percent reported they only had one symptom while more than 50 percent had 10 or more symptoms at the time[18]. Symptoms may include speech disturbances, motor problems, altered level of consciousness, sensory disturbances, cognitive challenges, and impairment in overall function.

In mild cases, the symptoms are often transient and may not require specific treatment. Many of these symptoms probably never come to the attention of health professionals. Moderate cases may require treatment. Severe, often chronic, conditions usually require specialised treatment.

FND symptoms are associated with high levels of physical disability, equivalent to people with multiple sclerosis or epilepsy, and even higher frequencies of psychological comorbidities than these disorders[19].

The most common symptom groups are:

Dissociative attacks (also called functional seizures or psychogenic non-epileptic seizures (PNES))
Dissociative attacks involve altered movements, sensations and states of consciousness that can look like epileptic seizures but are not caused by abnormal electrical activity in the brain.

Symptoms may include:

- Excessive movements of the limbs, trunk, and head
- Altered/loss of consciousness
- Feeling of lack of control of the body while conscious
- Reduced or absent speech volume (dysphonia/aphonia), altered articulation (dysarthria) or vocalisations (yelling, crying)
- Memory loss

These patients very commonly describe feelings of dissociation during, and sometimes before, the attack. Dissociation involves feeling disconnected from the body (e.g., thoughts, feelings, and sensations) and/or disconnected from the immediate environment. People often describe dissociation as feeling spacey, zoning out, going away, being “not quite there”, or as though their brain has shut down. Some people experience warning signs before episodes, however for others they can seem to happen automatically. Commonly the attacks are triggered by emotionally overloading situations such as stress, feeling overwhelmed or sensory overload.

Functional movement disorders
A functional movement disorder means that there is abnormal movement or positioning of

part of the body. This can result in a range of distressing and disabling symptoms that can also be seen in organic movement disorders, such as:

- Tremor (shaking)
- Dystonia (abnormal posturing)
- Myoclonus (jerks)
- Tics
- Spasms
- Gait disorders
- Limb weakness

Sensory disorders
Sensory symptoms are an often-overlooked group of functional symptoms but can significantly impact a patient's everyday life. For example, going shopping or meeting up with friends in a cafe can involve bright lights, loud music, visual overload such as all the products lined up in rows on supermarket shelves, sudden noises, and crowds of people to name a few challenges. Common symptoms are numbness, sensory illusions such as pins and needles and visual symptoms including blindness. Associated symptoms, not included as core FND ones, can include fatigue and headaches and 'psychiatric comorbidities including depression, anxiety, panic disorder and post-traumatic stress disorder[18].

Historical perspectives of, and terminology for FND

Symptoms and symptom patterns described in patients with FND have varied throughout history, strongly affected by the socio-cultural context and the diagnoses which are "popular at the moment"[20]. Many health professionals feel that FND is becoming more common although it is not clear that this is the case. What may appear as differences in occurrence may be caused by changes in the diagnostic designations that have been used in different periods in history. Historical descriptions of FND show very similar rates to what we see in clinical practice today. Also, while cultural context can be significant for what we call functional disorders and how we perceive them, rates of FND appear to be remarkably similar internationally[13].

Historically, many clinicians have used alternative names including, "hysteria'' (referring to the ancient Greek idea that a wandering and discontented uterus was blamed for excessive emotions in females), "conversion disorder" (referring to Sigmund Freud's theory of a repressed memory being "converted" to a somatic symptom) or "psychogenic disorder'' (referring to an assumed aetiology) to describe the symptoms of FND. Other terms that are frequently used are 'medically unexplained symptoms' and 'pseudo-seizures' or 'psychogenic non-epileptic seizures' (PNES; for patients with dissociative attacks).

These labels are potentially offensive to patients as they reinforce patriarchal messages from health professional of inadequacy in the self-management of perceived elusive symptoms and perpetuate blaming the patient. The term 'functional', however, has shown to be the most acceptable to patients [21] and places these disorders at the interface between neurology and psychiatry.

Many patients have had symptoms for a long time and have seen multiple doctors and been told "there is nothing wrong" or "the tests are all normal". This can be a demoralising process for patients who may give up seeking help. Simply telling patients the name of their condition can be extraordinarily powerful, even when they have been previously given the diagnosis in

a “reading between the lines” fashion[22]. The reassurance of having a name for one’s condition, even a bad condition, should not be underestimated.

Often health professionals are too hesitant to give the diagnosis of FND, however in cases where the symptoms are transient and minor and the patient presents only seeking reassurance, it may occasionally be appropriate not to give a diagnosis of FND, and instead to reassure the patient that “mild symptoms like this usually get better quickly and don’t signify anything bad”.

Does feigning or malingering occur in FND presentations?

FND describes motor and/or sensory symptoms that arise from the voluntary motor or somatosensory nervous system but are experienced as involuntary[23]. When these symptoms occur, they can be frightening and confusing for genuine patients who know they are not voluntarily feigning these experiences. Therefore, the responses given by health professionals are critically important. FND is **not** due to feigning or malingering. Unfortunately, many health professionals still have these wrong and hurtful opinions. In a study by Lehn et al. (2019) for example 10 percent of a survey of 516 Australian health professionals reported doubting whether FND symptoms were ‘real’. Further, these authors report that many health professionals in Australia have poor self-knowledge about FND and are not confident in talking with patients about such a diagnosis[24]. It would be naïve to think that feigning and/or exaggeration of symptoms cannot occur in patients with FND, but it is not unique to FND, and it can occur in patients with other disorders as well[25].

In recent years many research studies have been published that help us to understand underlying mechanisms leading to functional symptoms and how this is different from feigning. The most impressive studies are functional imaging studies and physiological studies (such as the libet clock paradigm or testing of sensory attenuation), which clearly show a pattern of abnormalities in patients with FND that cannot be explained by feigning or voluntary exaggeration[26,27].

Prognosis of FND

In general, the prognosis of FND is poor, but for many people, can be improved significantly with treatment. The majority of patients with FND are left with symptoms in the long term. Studies of functional motor symptoms for example show complete remission in only 21.5% of patients[28].

For patients with dissociative attacks, the published studies are heterogenous, but estimates for obtaining attack-freedom were relatively similar, with estimates ranging from 30% to 50%[29]. Rates of attack-freedom did not appear to change significantly between studies with short follow up (around one year), and studies with longer follow-up (longest six years), suggesting that most spontaneous remission probably happens early, after which prognosis without intervention becomes fixed. In children, recovery rates are more favourable with estimates of about 70% achieving seizure remission[29].

Section 2: Assessment and Diagnosis

Recommendations	
**B**	Assessment of FND should consist of a good history and examination, looking particularly for positive signs of FND.
**A**	FND is a clinical diagnosis based on history an examination. If positive features of FND are present on assessment, the diagnosis can be made with confidence.
**B**	A diagnosis of FND should rest on positive physical signs, which demonstrate<br>Inconsistency between impaired voluntary movement and intact automatic movement and/or<br>Incongruency with structural neurological disease.
✅	Generally, a diagnosis of FND should be made by a neurologist, ideally with specific interest this area.

It is helpful to take a broad person-centred approach based on a biopsychosocial model of health to appreciate the complexity and diverse representations of each FND patient within their own life and illness experience. Several interacting biological, psychological, and social factors can cause vulnerabilities, triggers and maintaining factors that contribute to FND[17] as outlined in Table 1 below.

Factors	Biological	Psychological	Social
**Factors acting at all stages**	Other neurological diseases<br>History of previous functional symptoms	Emotional disorder<br>Personality disorder	Socio-economic deprivation<br>Life events and difficulties
**Predisposition (vulnerabilities)**	Genetic factors affecting personality<br>Biological vulnerabilities in the nervous system	Perception of childhood experience as adverse -<br>Personality traits -<br>Poor attachment / coping style	Childhood neglect or abuse<br>Poor family functioning<br>Symptom modelling of others
**Precipitants (triggers)**	Abnormal physiological event (drug side effect, hyperventilation, sleep deprivation)<br>Physical injury / pain<br>Anaesthesia/surgery	Perception of life event as negative / unexpected -<br>Acute dissociative episodes / panic attacks	Social stressors/interpersonal conflict<br>Bereavement
**Maintaining factors**	Plasticity in central nervous system's motor and sensory pathways leading to habitual abnormal movements -<br>Deconditioning -<br>Neuroendocrine and immunological abnormalities similar to those seen in depression / anxiety<br>Symptom-focussed attention caused by the symptom itself (vicious cycle)	Illness beliefs<br>Perception of symptoms as being irreversible<br>Not feeling believed<br>Perception that movement will cause damage -<br>Avoidance of symptoms -<br>Fear of falling<br>Health anxiety/concern about other potential diagnoses	Receipt of invalidity benefits<br>Involvement in legal compensation processes<br>Ongoing medical investigations and uncertainty<br>Excessive reliance on wrong and unhelpful information which reinforce beliefs that symptoms are irreversible and purely physical in nature

TABLE 1: THE BIOPSYCHOSOCIAL MODEL WITH POTENTIAL FACTORS THAT MAY CONTRIBUTE TO FND (ADAPTED FROM NIELSEN ET AL[30]).

Distinct factors will be brought into play at various times in the course of the illness, and the biological, psychological, and social factors interact.

As to the biological aspect, changes in brain function and brain structure have been shown in functional disorders. Changes are seen in the connection between parts of the brain dealing with emotions and parts generating movement, as well as decreased activity in parts that contribute to the feeling that movements are voluntary. Research has also shown that explicit (consciously controlled) motor patterns override automatic movement in people with functional motor problems, which makes movements more difficult[17]. Psychologically, both cognitive and behavioural conditions such as illness understanding and illness behaviour are significant[8].

Assessment

Barriers to an effective assessment in FND

Patients with FND often have poor experiences when seeking help[31]. Common reasons include:

- Not getting the chance to describe their symptoms, inadequate explanation of the cause of symptoms, and limited discussion of potential treatment.
- Feeling that symptoms were dismissed or disbelieved.
- Perception that the doctor was adamant on finding a psychological problem to pin their symptoms on.
- Not being given enough time.

When seeing a patient with suspected functional symptoms for the first time, it is important to be aware of these barriers and the fact that patients may have had unsatisfactory experiences with health professionals before and take this into account.

What are the most important aspects of history taking?

There is a therapeutic element to history taking[32]. The purpose of this is not only to obtain information, but also to build a therapeutic relationship with a patient. Some therapeutic suggestions for obtaining history include:

Cover all symptoms: Make a list of all the symptoms your patient has and also ask about fatigue, pain, sleep and concentration. Having a complete list of current symptoms helps a patient to feel heard and prevents symptoms ‘popping up’ later.

Ask about dissociation: Dissociative symptoms are common in panic attacks and persistent fatigue but can be experienced in isolation. They are especially common in patients with dissociative attacks and patients with sudden onset functional motor symptoms. Patients are rarely able/willing to describe these symptoms spontaneously, partly because they do not know which words to use, but also the fear that the clinician will diagnose them with a psychiatric disorder. Therefore, it may be necessary to ask direct questions to reveal these symptoms. A patient discussing dizziness may actually be describing dissociative

symptoms. Common descriptions that can be used to prompt patients are feeling like they are out of their body, in a dream, or a long way away.

Ask what the patient thinks may be wrong and what should be done: Does the patient have any thoughts on why they have their symptoms? What does the family/friend think? Does the patient think they have epilepsy or Lyme disease? Take note if you are aware of this to tailor your explanation to it later. The discussion becomes therapeutic when the patient or family experiences relief by getting their worst fears and concerns out into the open. Find out if the patient is actually concerned about trying to get rid of their symptoms, or did they only present because their GP referred them or to be reassured that the symptoms don't represent a specific diagnosis (brain tumour and MS are common worries)? What do they think will help? It is difficult to treat patients who are not motivated to improve.

What happened with other doctors: If the patient tells you their previous doctor told them that the symptoms were "all in their head", it could be an indicator that the patient may be sensitive to psychological questions.

Be mindful of 'psychological' questions: It may be tempting to dive into questions about depression, anxiety, or stress, however it is not always necessary. Questions about prior psychological trauma such as physical or sexual abuse are likely to be unnecessarily intrusive on the first assessment unless the patient specifically wants to discuss it. If these things need to be discussed, it is preferrable to wait until the patient has gained confidence in their treating team.

Can talking about FND or testing for it make the symptoms worse?

It is worth noting that discussing and examining the symptoms may transiently worsen them, since attention to symptoms worsens symptoms of FND, but that no long-term worsening is likely to be caused by this. What patients often struggle with is not being told what is wrong with them and being left in 'limbo'[33]. Giving a clear diagnosis can be therapeutic in itself and discussing the possibility of FND in a non-judgmental way can reduce a lot of the stigma and fears that this diagnosis is still often attached to. Also, showing a patient with FND their positive physical signs not only gives the health professional confidence in the diagnosis but can also be very powerful to show patients that their diagnosis is accurate and has the potential for reversibility[34].

Assessing a patient for positive symptoms of FND

There are many tests for functional neurological disorders due to its broad nature, and a discussion of all the individual tests is beyond the scope of these guidelines.

In general, one should look for inconsistencies in symptoms where function varies in different situations (positive symptoms).

Examples of inconsistencies in functional motor symptoms[7]:

- Walking, and then being unable to lift a leg off the bed
- Gesturing with hands while speaking when unable to voluntarily move the hands
- Hoover's sign in functional limb weakness
- Entrainment in functional tremors

Examples of incongruity in dissociative attacks (compared to epileptic seizures)[7]:

- Very long duration is more common in dissociative attacks
- Resisting eyelid opening is more common in dissociative attacks

Should you perform additional investigations in patients with FND?

A diagnosis of FND should be based on history and examination findings. The diagnosis can be made solely on positive clinical signs and is not a diagnosis of exclusion. In some situations though, it may be appropriate to perform tests to rule out other differential diagnoses or look for comorbid conditions. In this circumstance, we recommend telling the patient that the probable diagnosis is FND and that the tests are to make sure that other (underlying or coexisting) conditions aren't being missed, as would be done in other instances where there is a strong working diagnosis. An analogous discussion would be the patient with migraine headaches who is sent for a scan because of soft red flag symptoms.

Diagnosis

Are there diagnostic criteria for a Functional Neurological Disorder?

In 2013, the term Functional Neurological Symptom Disorder/Conversion Disorder was adopted by the Diagnostic and Statistical Manual of Mental Disorders. In the current Fifth Edition, Text Revision (DSM-5-TR) FND is classified as "Functional Neurological Symptom Disorder (Conversion Disorder)" in the chapter "Somatic Symptom and Related disorders, code F44.X"[35]. According to these criteria, a diagnosis of FND should be made in an inclusionary manner by identifying neurological signs that are specific to FND.

**Diagnostic Criteria for Functional Neurological Symptom Disorder (Conversion Disorder) according to DSM-5-TR**	
**A**	One or more symptoms of altered voluntary motor or sensory function.
**B**	Clinical findings provide evidence of incompatibility between the symptom and recognized neurological or medical conditions.
**C**	The symptom or deficit is not better explained by another medical or mental disorder.
**D**	The symptom or deficit causes clinically significant distress or impairment in social, occupational, or other important areas of functioning or warrants medical evaluation.

TABLE 2: THE DSM-5-TR CRITERIA

In earlier versions of the DSM, the term conversion disorder was used instead of functional, implying that psychological abnormalities are the sole cause of these disorders. Furthermore, in the latest version the presence of psychological stressors as a criterion has been removed, paving the way for a biopsychosocial diagnostic model.

In the 11th revision of the International Classification of Disorders (ICD-11) by the WHO, FND is classified as "dissociative neurological symptom disorder" in the chapter "Mental, Behavioural or Neurodevelopmental Disorders, code 6B60.X," as well as in the chapter

Lehn A, *et al. BMJ Neurol Open* 2025; 7:e000970. doi: 10.1136/bmjno-2024-000970

"Diseases of the Nervous System, code 8A0X" under the term "movement disorder for parkinsonism, dystonia, and tremor"[36].

How is the diagnosis of FND made?

In most cases FND can be diagnosed accurately and with confidence based on positive signs. The two hallmark features of FND on examination are inconsistency and incongruity[7].

Inconsistency can for example be a tremor that is present one moment, but then settles down when distracted (or that can be 'entrained' to another frequency). Incongruity describes incongruity with recognised neurological diseases and the neuroanatomy of the human body. To pick this up with confidence requires knowledge of typical presentations of organic disorders and of neuroanatomy. If the diagnosis of FND is approached this way, it not only gives the health professional and patient confidence in diagnosis but can then also set the patient up well for subsequent treatment.

Who can make the diagnosis of a Functional Neurological Disorder?

The spectrum of FND represents a wide range of severity, comprising sub-clinical and mild deficits through to severe disabling disorders. The symptoms are often similar to those of other neurological conditions, and therefore a neurologist is best placed to make the diagnosis. As the field of neurology expands, some neurologists sub-specialise in particular areas, such as movement disorders, epileptology, or paediatric neurology. Therefore, ideally, the differentiation between a functional or non-functional neurological symptom should be made by a neurologist with specific expertise in the relevant area.

However, access to a neurologist is often limited, especially in more rural parts of Queensland. In those cases, and depending on the patient's key symptoms, it is appropriate for other specialists (e.g. rehabilitation physician or psychiatrist) with knowledge in this area to make the diagnosis of a Functional Neurological Disorder. Also, the possible diagnosis may be raised with the patient in primary care or by an allied health professional. In these situations, having knowledge about how the diagnosis is confirmed greatly aids subsequent management[37].

Differential diagnosis and comorbidity

Recommendations	
**B**	In the right hands the misdiagnosis rate for FND is rare.
**B**	Comorbidity between FND and non-functional disorders is common (e.g., dual diagnosis of epilepsy and dissociative attacks).
**B**	Detecting comorbidities may be useful in treating the patient but the presence of certain comorbidities (such as mental health disorders) should not be the reason for making the diagnosis of FND.

How likely is a diagnosis of FND to be correct?

In clinical practice, there is a fear of misdiagnosis of FND, however, studies have repeatedly demonstrated low rates of misdiagnosis. A systemic review of historic studies of FND showed a misdiagnosis rate of about 4% since 1970[38]. In a more recent large cohort study of

patients with Functional Neurological Disorder diagnosed in neurology clinics and followed up for 18 months, the misdiagnosis rate was only 0.4%[12]. This is much lower than the misdiagnosis rate in the vast majority of other neurological disorders[39].

Does a diagnosis of FND rule out other underlying disorders?

A proportion of patients with FND have more than one diagnosis causing similar symptoms, for instance, epilepsy and dissociative (non-epileptic) attacks; multiple sclerosis and functional limb weakness; idiopathic intracranial hypertension and functional visual symptoms[40,41].

It is important to be aware of the high rates of comorbid psychiatric disorders in FND, because these underlying disorders might require different or additional treatment strategies[13]. However, it is just as important to realise that many with FND do not suffer from underlying mental health issues and that psychiatric comorbidity is also very common in other neurological diseases.

Section 3. Treatment and management

Most patients with functional disorders benefit from treatment. For patients with mild and transient symptoms making an accurate diagnosis and giving an appropriate explanation might be enough. The explanation and patient education should be started by the diagnosing specialist and supported by the GP and allied health professionals.

When an explanation alone is unsuccessful patients can benefit from brief and tailored treatments of their functional neurological symptoms. For patients with functional movement disorders this would typically be with a physiotherapists or occupational therapist. For patients with dissociative attacks a psychologist should likely be involved.

Some patients with severe and intractable functional symptoms will require input from multidisciplinary services. While there are some services available through the public and private system, there is still a large unmet need for high-level multidisciplinary services in this area.

The Role of the General Practitioner

Recommendations	
✅	The patient's GP is informed of the diagnosis and provided information on how to best support the patient in ongoing management.
✅	The GP-patient relationship is recognised as pivotal as a way of providing care in the longer term. They should have access to specialist advice and be given opportunity for continuing professional development in the management of FND.

Naturally, there is an emphasis on assessment and diagnosis of FND yet often long-term management is required – and the GP plays an important role in this. Being able to get this support is complicated by very limited access to public services and private services that come with a financial burden for patients perhaps already in financial stress due to their disorder. The ongoing patient journey can be a lonely one requiring self-advocacy and-agency to keep up with new research and practices which could improve one's quality of life as well as educating clinicians simultaneously who may know less about FND.

Patients may also find themselves as their own case managers, facing siloed rather than multidisciplinary treatment options, a rather daunting experience when first diagnosed. Having to take on the burden of administrating one's own treatment plan can flare FND symptoms, slowing down any opportunity for improvement. A supportive and proactive GP can make a huge difference. Specialists should not underestimate the effectiveness of such a therapeutic relationship and should encourage patients to seek out such support. Likewise, they should include GPs as part of a multidisciplinary team with the opportunity for ongoing professional development.

It may be challenging for patients to find a sympathetic GP with knowledge in this area given the community demand for their services and the pressures on their time to upskill in FND management if they are even interested to do so. This situation may be particularly relevant in regional and rural locations where even more basic challenges existing in attracting and retaining GPs. Access to telehealth consultations with more experienced GPs may be one

way forward, so patient needs are met but the less experienced GP is also mentored in how to manage FND.

The role of the neurologist

Recommendations	
✅	The neurological assessment can be seen as the start of the treatment in FND, not just a prelude to diagnosis.
**B**	Effective explanation of a diagnosis of FND can alter key beliefs in patients and foster helpful behavioural changes
✅	Neurologists have a role in triaging to different types of evidence-based treatment.

What is the role of neurological assessment in the treatment of FND?

Neurological assessment in FND can be both diagnostic and therapeutic. The primary aim of the consultation regarding symptoms that may be functional is to establish the diagnosis. Ideally a Neurologist is involved with making and/or confirming the diagnosis of FND, however this may not always be practical or necessary.

After making the diagnosis, a good explanation of FND is important, and can markedly improve prognosis[22]. Explanation should include the nature of the symptoms in terms that make sense to the patient. Explanation and demonstration of the key feature that symptoms worsen with attention and improve with distraction can help set the background for future treatment by other health professionals, as this understanding is critical to most symptom-treatment exercises.

Frequently, patients will have concerns that they have a specific organic cause for their symptoms (e.g. MS or a brain tumour). These concerns should be specifically addressed in the explanation, with reference to how the diagnosis of FND was made and the incompatibility of the features seen with the condition the patient is concerned about. Investigations to further reassure patients are rarely necessary after an empathetic discussion of these concerns but can be helpful in some situations when done appropriately and with a clear focus.

For some common symptoms, neurologists may be able to recommend and teach some exercises that patients can do at home to begin treating their symptoms before seeing other health professionals. Examples could be simple distraction exercises for gait difficulty, practicing Hoover's test as a way of generating movement to improve lower limb weakness, manoeuvres such as isometric contraction or self-entrainment for stopping tremor, and sensory grounding exercises (e.g. the 5,4,3,2,1 exercise) for premonitory symptoms of dissociative attacks.

How can relapses of FND be prevented and pre-emptively treated?

Even if patients fully recover from FND in the short term, the majority of patients will remain vulnerable to developing flares of functional symptoms in the future[42]. It is very helpful to manage expectations for short-and long-term outcomes appropriately. With good multidisciplinary management many patients can improve from their functional symptoms, and some can even have full resolution of their symptoms, but it helps to work on a relapse

management plan from an early stage. This includes developing a good understanding of triggers and early warning signs as well as the developing of a 'toolbox' of techniques that patients can use to manage symptoms better in case of future flares. Several resources are available on the internet to help with this, for example the 'FND workbook' that has been developed by the Australian FND Network (fndaustralia.com.au).

Are there any materials that a neurologist or other healthcare professional can refer to?

A good understanding of the diagnosis is key in the treatment of FND and patients should be encouraged to further review educational materials on websites such as fndaustralia.com.au and www.neurosymptoms.org.

The fndaustralia.com.au website has written information about FND and its management, but also several educational videos that explain key aspects of the diagnosis and management of FND. Furthermore, the Australian FND Network has developed a workbook (which can be downloaded from the website) to help guide patients on their recovery journey. It is a tool which can help patients to better understand Functional Neurological Disorders and where they can record what they have learnt and accomplished from the various therapies.

The role of the physiotherapist

Recommendations	
**A**	Physiotherapy management should include facilitating normal movement, retraining normal movement, addressing secondary changes and education (including role of physiotherapy, activity pacing and long-term self-management of symptoms).

What is the role of the physiotherapist in the treatment of FND?

There is increasing evidence for physiotherapy management of FND (specifically for functional movement disorders) in both inpatient and outpatient settings, particularly when working and collaborating in multidisciplinary teams. Synthesis of current research is out of scope for this publication but there are several excellent summaries available in the published literature, such as Glen Nielsen's article 'Physical treatment of functional neurologic disorders' in the Handbook of Clinical Neurology[43].

What are the physiotherapy assessment options for various functional symptoms?

Subjective Interview

The aim of physiotherapy assessment in FND is to:

- Gain a holistic picture
- Set goals for physiotherapy treatment
- Gain rapport
- Gauge the patient's understanding and level of confidence in diagnosis

History of Presenting Condition

- Identify all symptoms
    - Biopsychosocial triggers / aggravating factors
    - Biopsychosocial easing factors
    - Behaviour of symptoms over 24 hours (duration, frequency)
- Who provided diagnosis
- Impact on activities of daily living
- Explore the patient's understanding of and level of confidence in the diagnosis already given
- Current Level of function

Past Medical History

- Medications

Social History

- Occupation
- Home support
- Home environment
- Support network
- Existing funding/services

Previous level of function

- Mobility status
- Hobbies

Objective assessment

The assessment should emphasise activity performance and functional ability (e.g., posture, transfers, mobility, gait pattern and upper limb function) over impairment (e.g., muscle strength and coordination), as impairment testing is usually inconsistent with functional ability. This is likely to unveil "positive signs" that may help consolidate the diagnosis. It is recommended that novice learners / those not comfortable with assessing FND complete a full neurological assessment.

Suggestions for isolated symptoms

Weakness: observe voluntary movement of the affected limb, then trial with contralateral opposition e.g., Hoover's sign. Fluctuating weakness with distraction or dual tasking is a potential indicator of functional contributions. In patients who can mobilise, inconsistent patterns e.g., apparent foot drop on forward ambulation but active ankle dorsiflexion when walking backwards or inability to perform standard sit-to-stand despite being able to reciprocally descend stairs, may also be used.

Tremor: ask the patient to complete or copy a competing alternating movement with the non-affected limb if able. Alternatively, engage the patient in complex cognitive dual tasking e.g., counting or language. Entrainment (synchronising of tremor to intentional movement) or distraction are potential indicators of functional contribution.

Lehn A, *et al. BMJ Neurol Open* 2025; 7:e000970. doi: 10.1136/bmjno-2024-000970

Dystonia: trial contract-release of affected agonist and antagonist muscles and competing sensory input e.g. TENS. Unusual responses (e.g. complete and rapid resolution of posture with TENS followed by immediate return on cessation) are potential indicators of functional contributions.

Functional gait disturbance: trial impacts of distraction e.g. upper limb movements and complex gait tasks such as tandem walk. Improvement of gait stability and coordination with complex dual tasking is a potential indicator of functional contributions.

Outcome measures

Due to the heterogenous nature of FND, it is important to consider a combination of subjective and objective measures. Selection of which measures should be reflective of your service model, whether it will be used for future research purposes and the symptom phenotypes (i.e.. 10m walk test for functional gait disturbance; 9 hole peg test for functional upper limb tremor; laterality of affected limb).

The Simplified Functional Movement Disorder Rating Scale should be used to capture all motor phenotypes. Other potentially useful subjective outcome measures include Clinical Global Impression Scale, Functional Mobility Scale, Disabilities of the Arm, Shoulder and Hand, Brief Illness Perception Questionnaire, Modified Fatigue Impact Scale and EQ-5D-5L.

What are the physiotherapy treatment options for functional motor symptoms?

This section has been adapted from the Physiotherapy Consensus Recommendations[30] – see Resources for further guidance.

Tremor: trial competing movements (i.e.. Giving limb another meaningful purpose), distraction and intentionally amplifying the tremor to assist in recognition of control.

Weakness: encourage early weightbearing and avoid prolonged rest. Dual tasking or skill practice with distraction may be beneficial, as may the use of novel surfaces e.g. foam.

Dystonia: bring attentional focus to external factors rather than the dystonic limb and address any pain or protective factors which may be leading to habitual positioning. Encourage routine sensory input, mirror box therapy and consider trial of functional electrical stimulation if tolerated. Dystonia is commonly associated with persistent pain (refer to FND & persistent pain section).

Functional gait disturbance: Trial novel walking patterns e.g. increasing speed, changing environment, deliberate ‘moon walking’, dual-tasking, gradually weaning UL support and distraction. Taking a video of patients on their own phones may be helpful given the proprioceptive disruption which is a hallmark of FND.

Treatment for FND should be goal orientated in collaboration with the patient and largely focuses on facilitating self-management of FND symptoms to maximise a person’s rehabilitation. This includes education about FND, identifying triggers, energy conservation education, pain management, carer and family education, anxiety management and creation of a relapse prevention plan.

The role of the occupational therapist

Recommendations	
✅	Patients with FND often have problems coping with daily life, and therefore, occupational therapy could be seen as a natural fit for treatment.

What is the role of the occupational therapist in the treatment of FND?

Occupational therapists, with their training in both the physical and mental health domains and the focus on function and participation, contribute greatly to the multi-disciplinary team and biopsychosocial model of care frequently used in people with FND[44]. Occupational therapists assess the impact of symptoms on function and the need for strategies, care and/or equipment to support engagement in meaningful daily activities (personal care, domestic activities, productivity and learning, community or leisure activities). Intervention involves goal setting, education, activity analysis and grading tasks, facilitating coping/problem solving strategies, retraining abnormal movement patterns during daily activities, supporting self-care, self-awareness, and self-management (sleep, fatigue, pain, stress, memory/cognition, energy conservation, distraction, interception, sensory modulation) and facilitating access to patient's homes, schools, workplaces and community environments. Another important aspect of treatment is risk management (supported positive risk taking) and relapse prevention planning.

What are the occupational therapy assessment options for various functional symptoms?

A range of occupation-based assessments have been identified to support the clinician when working with people with FND including the Canadian Occupational Performance Measure, The Occupational Self-Assessment, The Assessment of Motor and Process Skills and The Model of Human Occupation Screening Tool. Assessments of physical function, sensory processing and observations during the performance of daily tasks are also recommended.

What is the current evidence for occupational therapy (OT) intervention?

Overall, there is limited evidence for specific OT interventions for clients with FND. However, there are varying levels of evidence available that support OT intervention as part of inpatient and outpatient MDT programs as individual and group treatment sessions.

The Occupational Therapy Consensus Recommendations for FND paper by Nicholson et al, 2020 outlines the current evidence and consensus recommendations for the OT role in the assessment and treatment of individuals with FND[44]. It is a great place to start when researching your approach for a patient with FND.

Below is a summary of what the available evidence and recommendations identified as OT specific assessments and treatments for patients with FND:

Assessment and goal setting

Standard occupational therapy initial assessment tools should be used to obtain information about a person's previous and current level of function, experiences, home, and social

environments. Nicholson et al recommend gaining a picture of their 24-hour routine as this can be helpful to uncover triggering factors.

Home, workplace and/or school visits can be helpful to assess function and access. However, not all services have provision for this and a detailed history from the client and family will be necessary to help guide intervention and equipment use if required.

Goal setting tools such as the COPM, CMOP-E and MOHO, are recommended over standardised outcomes measures to encourage focus on functional improvement and deter focus on specific symptoms/measurements e.g. strength, pain, dexterity.

Sensory processing difficulties are commonly reported by patients with FND and can contribute to avoidance and limit function impacting on length of recovery. It therefore should be considered as part of the initial occupational therapy assessment and information gathering process. For further assessment, The Sensory Preference Checklist or The Adult/Adolescent Sensory Profile 2 are recommended tools.

OT Treatment

The role of occupational therapy in the treatment of FND focusses on reengagement in a person's normal activities of daily living. Interventions include motor retraining and desensitisation, energy conservation principals, graded return to activities, sensory modulation, vocational rehabilitation, psychosocial intervention and education to support self-management. Engaging the patient and their family/carers in problem solving and establishing intervention strategies to support function is particularly important.

Motor symptoms

Motor symptoms of FND often include abnormal movement, weakness, dystonia or tremor. There are several techniques that clinicians can use to try and facilitate normal movement patterns. Many of these techniques are outlined in the Nicholson et al 2020 Consensus article and include distraction through verbal activities/conversation while completing a task, eliciting normal movement though use of "FND positive sign tests" and even use of electrical stimulation in conjunction with a functional activity. Graded Motor Imagery can also be useful in managing pain symptoms of FND[45]. Splinting/restricting the effected limb is to be avoided as it may result in learned non-use and other negative side effects.

Sensory symptoms

Hypersensitivity to light, touch, sound and movement are often reported by patients with FND as outlined in the OT Consensus Recommendations.

Occupational therapy treatment focuses on increased participation and function through:

- Providing support and education to help patients and their family members/carers understand the influence of a heightened nervous system on their sensory system and the interplay between the two which can contribute to reduced function.
- Facilitating patient (and family/carer) awareness of their sensory profile scores and sensory preferences and how this can be a limiting factor to their functional recovery.
- Graded exposure to triggering sensory environments/inputs, the use of compensatory strategies such as sunglasses, headphones, protective postures (gradually minimised) and establishing a sensory toolbox of strategies that support an individual's regulation are all recommendations that contribute to increased participation and function.

Cognition

Individuals with FND may also present with cognitive difficulties. This can be due to several factors including medication side effects, pain, fatigue, anxiety and other mental health

comorbidities. It is important to first discuss with the team whether this is a symptom of an individual's FND or related to an underlying issue. Managing these cognitive symptoms usually involves addressing the contributing factors, educating the individual and encouraging structure and routine to manage cognitive overload. It is often not helpful to complete formal cognitive testing unless specifically required.

The role of the speech pathologist

What is the role of speech pathologists in the diagnosis and management of FND?

As with other manifestations of FND, the first step in treatment is a correct diagnosis followed by a good explanation in terms that the patient can understand, emphasing the important aspects of FND treatment (trigger identification and management and the role of attention in worsening symptoms and distraction in improving them). This can be done by multiple members of the team, but for relevant symptoms, can be either led or reinforced by the speech pathologist[46].

Speech pathologists are trained in assessing disorders of language, speech production and swallowing. For patients where these are the main symptoms of FND, they may be the best placed member of the healthcare team to diagnose the origin of these symptoms as functional, and to demonstrate to the patient the signs that lead them to this diagnosis. As with other symptoms seen in FND, signs of functional communication or swallowing disorders are inconsistent either with symptoms, over time, or with distraction. Some signs are pathognomonic of functional disorders.

Isolated communication and swallowing symptoms may sometimes be appropriate to be managed by a speech pathologist alone, but if progress is sub-optimal, or if there are other symptoms, engagement of other members of the multidisciplinary team is recommended.

What are the speech pathology assessment options for various functional symptoms?

General speech pathology assessment features

The following features may be signs of a functional communication disorder:

- Symptoms vary across assessment tasks
- Symptoms become worse when the patient is aware that they are being assessed or if they are told an assessment task may be difficult
- Symptoms worsen with particular people, situations or other stimuli (e.g. noise, smells)
- Symptoms are reduced or resolved during casual conversation or when enthusiastic about topic
- Symptoms resolve after 1-2 therapy sessions

Voice

The vocal cords should be examined first to rule out any structural or neurological cause for the voice disorder. Routine perceptual voice assessment can then be conducted, as well as assessing voice in a range of speech tasks (e.g. isolated sounds, automatic phrases, familiar

phrases, complex conversation). More severe voice symptoms may be seen when emotional topics are discussed. Signs of functional voice disorders include: incongruency between voice symptoms and expected clinical presentation based on physical examination of vocal cords, inappropriate vocal quality for age/gender, inconsistency in vocal quality across tasks and exaggerated mouth, face or neck movements associated with voicing attempts (which may resolve with the return of normal voice). Using strategies to achieve reversal of symptoms (i.e. eliciting normal voice production) is another powerful assessment tool (and provides encouraging information about potential for treatment). Reports of globus should also be monitored, as this often resolves in line with the resolution of other vocal symptoms[46].

Fluency

A range of typical fluency assessment tasks can be used e.g. automatic speech tasks (such as counting, days of the week), conversation, reading aloud (repeat the same passage multiple times), speaking with delayed auditory feedback or in the presence of white noise, singing. Signs of functional fluency disorders include: onset of acquired stuttering as an adult in the absence of aphasia, apraxia of speech or dysarthria; no periods of fluency on automatic speech tasks; stuttering may be seen on all sounds in words; continued stuttering during tasks that would typically result in reduction in stuttering (e.g. singing, reading the same passage aloud multiple times, speaking in the presence of white noise or delayed auditory feedback, miming words/sentences/reading aloud); inconsistent pattern of dysfluency; associated struggle behaviours (e.g. facial grimacing, head jerking, hand slapping, eye squinting, neck extension); rapid resolution of symptoms following brief intervention[46].

Articulation

Oromotor assessment should be conducted as well as recording speech samples. Signs of functional articulation disorder include: oromotor function being inconsistent with speech presentation; speech sound errors being atypical and associated with unusual and exaggerated mouth/tongue/lip movements or other unusual prosodic features; errors may be consistent but limited to particular sounds and some speech sounds may be produced with significant variability. Speech sound errors may be associated with developmental errors (i.e. ‘read’/’wead’)[46].

Language and cognitive-communication

Typical speech pathology assessments may be used. Signs of functional language and cognitive-communication disorders include: incongruency between language/cognitive-communication skills and expected clinical presentation based on imaging, and significant variability in symptoms over time or across different situations[46].

Swallowing

Bedside swallow assessment, instrumental swallow assessment [e.g. fiberoptic endoscopic evaluation of swallowing (FEES) or videofluoroscopic swallowing study (VFSS)], and validated self-report measures of dysphagia severity may be used[47]. It is important to rule out any other disease or structural/physiological abnormalities as the cause for dysphagia. Signs of functional dysphagia include: being able to spit but not being able to control anything in the mouth, and being unable to swallow food/fluids despite normal saliva management. Common symptoms of functional dysphagia include: coughing, choking sensation, globus, painful swallowing or feeling difficult to swallow[46].

What are the speech pathology treatment options for various functional symptoms?

Voice

Explain to the patient that voice can be brought under their control (and this is the goal of treatment). Use techniques to generate voice such as natural/reflexive behaviours with sound (e.g. cough and clear throat, yawn-sigh, easy onset with prolonged speech sounds e.g. /mmm/), playful pre-linguistic vocal sounds (e.g. blow raspberries while voicing), automatic utterances (e.g. counting, sing favourite song), redirecting attention (e.g. headphones to alter auditory feedback, talking while engaging in other body movements e.g. walking, jumping), and physical manoeuvres (e.g. circumlaryngeal massage with concurrent vocalization, for treatment of musculoskeletal tension). Traditional evidence-based dysphonia treatment may be effective e.g. vocal function exercises, semi-occluded vocal tract exercises, resonant voice exercises. The above direct therapy techniques along with indirect approaches (such as education and vocal hygiene) can be effective. If there is a link between vocal symptoms and any ongoing psychosocial issues, it is important for the patient to be aware of this association and have strategies in place to manage them (especially to achieve generalisation of treatment gains beyond the therapy setting)[46].

Fluency

Educate the patient about the importance of forward airflow and reducing muscle tension to achieve smooth speech. Use visualisation to facilitate smooth speech (e.g. thinking about a surfer riding a wave). Use developmental stuttering therapy techniques e.g. slow rate, easy onset, connected speech[46]. A typical treatment hierarchy may be used e.g. start with word repetition and progress to short sentences and conversation[48]. Aim to eliminate secondary movements by using distraction (and phase this out as fluency improves) e.g. lying on their back while talking, finger tapping while talking, squeezing a ball while talking, playing music through headphones while talking. The speech pathologist should also liaise with the patient’s mental health professional to manage psychological distress or anxiety[46].

Articulation

Traditional treatment approaches used for developmental and neurological articulatory disorders may be effective to treat functional speech impairments. Some specific treatment strategies for functional articulation disorders may include the reduction of musculoskeletal tension, implementing distraction techniques, introduce mindfulness skills during oromotor tasks as a way of maintaining focus on easy, smooth movements and the use of nonsense words, syllable repetitions or singing as a way to demonstrate potential for ‘normal‘ function and distraction from abnormal sounds[46].

Language and cognitive-communication

Symptoms may resolve once other functional symptoms are treated. Compensatory strategies may be taught if language and cognitive-communication difficulties persist e.g strategies for word retrieval, reminders to correct grammatical errors in speech[46].

Swallowing

Explain normal swallowing to the patient (including that the pharyngeal stage of swallowing is an automatic process, that the cough is a protective function, that it is normal for everyone to penetrate/aspirate to an extent, and that choking usually only occurs with solid unchewed pieces of food). Use the patient’s instrumental swallowing images to provide a visual demonstration of their swallowing. Acknowledge any anxiety or life stressors that are contributing to symptoms (it will often be appropriate to liaise with the patient’s mental health

Lehn A, et al. BMJ Neurol Open 2025; 7:e000970. doi: 10.1136/bmjno-2024-000970

professional about this also, particularly around cognitive behavioural therapy strategies that may be useful). The patient may also receive treatment from a mental health professional for any comorbid mental health disorders. Reinforce normal dining etiquette (e.g. sit upright for meals, don't talk while eating, make sure food is chewed well and the mouth is not overfilled). Any muscle tension related to swallowing should be addressed (e.g. in the head, neck or jaw). Develop a hierarchy of difficulty with food/drink from the patient's perspective and use desensitisation and graded exposure to target anxiety around swallowing[46]. Consider referrals for dietetics and nutritional input to ensure the person is able to consume enough nutrients to be able to function.

Not included: Assessment and treatment options for functional cough and vocal cord dysfunction, and foreign accent syndrome (see Baker et al. 2021 for information on these functional disorders).

The role of the psychologist

Recommendations	
**A**	Cognitive Behaviour Therapy (CBT) alone compared to standard medical care has proven to be beneficial in the treatment for FND.
**B**	Provide evidence-based psychological therapy for the driver of the problem and/or comorbid psychological condition - e.g., Dialectical Behaviour Therapy for Borderline Personality Disorder, trauma-focused psychotherapy for PTSD.

What is the role of the psychologist in the treatment of FND?

Evidence for effective psychological intervention for treating FND is emerging, particularly since the change in diagnostic criteria with DSM-5. Psychological therapy is recommended for people with FND to target factors contributing to the development and maintenance of symptoms. Common mechanisms identified in the literature include dissociation, changes in emotional processing, and sense of agency, difficulty regulating emotions, and alterations in interoceptive awareness.

There is now substantial research evidence investigating the use of cognitive behaviour therapy in FND, but not all studies are positive. There is limited evidence for the use of expressive psychotherapies in this population and prospective trial evidence is urgently needed.

What is psychology's role in diagnosis?

There has been an important shift in diagnosis of 'Functional Neurological Symptom Disorder (FNSD)' (as it is called in DSM-5) to one of 'rule-in' criteria, relying on the presence of positive signs that signal neurological symptoms that are incompatible with known neurological or medical disease. The requirement of identifying preceding stressors and the exclusion of feigning have been dropped from the DSM-5[49]. However, Criterion C still requires the exclusion of other mental health disorders that may explain the current symptoms[49]. As such, psychologists should conduct a psychiatric differential diagnosis for patients diagnosed by neurologists/medical doctors with functional neurological symptoms. Differential diagnostic considerations include those in the Somatic Symptom and Related Disorders category, such as Somatic Symptom Disorder (SSD), Illness Anxiety Disorder and

Lehn A, et al. BMJ Neurol Open 2025; 7:e000970. doi: 10.1136/bmjno-2024-000970

Factitious Disorder. Functional neurological symptoms can occur in the presence of another neurological or medical condition (e.g., non-epileptic seizures / dissociative attacks and epilepsy). Other psychiatric conditions in addition to FNSD need to be considered in these cases (e.g., psychological factors affecting a medical condition, SSD, or an adjustment or anxiety disorder). Several psychiatric conditions in other diagnostic categories also share features with FNSD. For example, dissociative disorders, Post-Traumatic Stress Disorder, and Tic Disorder all manifest with a high somatic burden (e.g., tremors, fatigue, weakness, or tics) and may be comorbid diagnoses or may better account for the symptoms in question[49]. When symptoms of one or more psychiatric disorders are identified, clinical formulation of the mechanisms of the symptoms will help determine the primary psychiatric condition and any comorbidities (see Table 4).

Brief clinical examples	
Ms Y (28F) was referred to an outpatient neurology clinic following a workplace accident that resulted in a minor head injury. Alongside frequent headaches, she described persistent 'brain fog'/vagueness, memory loss, difficulty sustaining attention, dizziness and pins and needles in her arms and legs. More recently, her vision has been blurred intermittently. Testing, including imaging, revealed no abnormalities and her clinical neurological examination was inconsistent.<br><br>Clinical interview further revealed that while worse since her accident, most symptoms had been present since late adolescence and have waxed and waned over time in relation to life stressors. She strongly endorsed feelings of unreality and being detached from her body, was emotionally numb and had difficulty expressing her emotional experience.	Diagnosis: Depersonalisation/ Derealisation disorder.

TABLE 3: BRIEF EXAMPLES OF DIFFERENTIAL DIAGNOSTIC CONSIDERATIONS FOR PATIENTS PRESENTING WITH FUNCTIONAL NEUROLOGICAL SYMPTOMS.

How is CBT used in the context of FND?

The highest level of evidence is for Cognitive Behaviour Therapy (CBT)[8]. This is likely due to the structured and short-term nature of therapy and the ease at which it can be manualised. CBT focuses on reducing symptoms by targeting cognitive (i.e., illness beliefs, symptom-focussed attention) and behavioural (e.g., overcoming avoidance, emotion regulation) factors which may be perpetuating symptoms. As CBT has relatively little emphasis on emotional and interpersonal processes and is focussed on the here and now, it may make CBT more palatable for some patients (i.e., alexithymia or highly avoidant).

CBT in FND populations has shown to provide longer event free period compared to previous six months, better health related quality of life, psychosocial functioning, less psychological distress, less somatic symptoms, greater reported improvement (patient and clinician) and greater satisfaction.

The role of the psychiatrist

Recommendations

Lehn A, et al. BMJ Neurol Open 2025; 7:e000970. doi: 10.1136/bmjno-2024-000970

✅	Consider a referral to a psychiatrist for additional diagnostics and treatment of psychiatric comorbidities in patients with FND.

What is the role of the psychiatrist in the treatment of FND?

While the diagnosis of FND is ideally made by a neurologist, it has traditionally been supported by a psychiatrist. The diagnosis is best made purposefully on a ‘rule-in’ basis, observing positive signs elicited by the neurologist. While a psychiatrist may also perform a physical examination and elicit positive signs, their most important contribution to diagnosis is in obtaining a detailed history that may further support the neurologist’s diagnosis. From a psychiatric perspective, FND has traditionally been conceptualized as one of the many downstream consequences of trauma. While this applies to a significant subgroup of patients, it may not be applicable in every case. While a remote history of trauma is thought to prime the patient to a risk for FND; and a psychological (or physical) trauma commonly (but not invariably) immediately precedes the disorder’s onset, this history is no longer needed to satisfy a diagnosis of FND. Nevertheless, such a history is supportive of the FND diagnosis when present.

Once a diagnosis is made, the psychiatrist can continue to play a valuable role in ongoing treatment in a number of ways. Psychoeducation, identification and treatment of comorbid psychiatric conditions and liaison with family and other health professionals involved in the patient’s care is a central role of the psychiatrist. Assessment for, and if appropriate, provision of psychotherapy may be a useful element of continuing patient care. This not only relates to improving the patient’s symptoms, but in the substantial group with residual symptoms, supporting the patient and their family in their adjustment to continuing disability and addressing stigma that can be associated with the condition.

When is pharmacological treatment indicated in FND?

Psychotherapy and pharmacotherapy in FND are both an important part of the history of psychiatry. Unfortunately, the development of evidence for their use has been, and continues to be, dogged by a number of obstacles. Caseness, symptom heterogeneity and complex comorbidity, not only in clinical presentation, but also trial design, have limited the evidence base for clinicians to draw upon.

The identification and treatment of co-morbid psychiatric diagnoses such as mood or anxiety disorders with evidence-based psychotherapy and or pharmacotherapy is the first responsibility of the consulting psychiatrist.

While a few studies have examined the use of SSRIs and SNRIs in FND, evidence does not yet support the prescription of these agents to directly target FND symptoms.

In the absence of a co-morbid Axis I diagnosis however, a psychiatrist may still have an important role in patient care. As a ‘last line of defence’ in general hospital psychiatry, treatment can occur once a diagnosis is confirmed. Careful history taking with a recognition of salient developmental issues, current psychological conflicts, recognition of immediate precipitants to the presentation and observation of a waxing and waning pattern of symptoms may be central to supporting the diagnosis, formulation and development of a tailored treatment plan. It is reasonable practice for an assessing psychiatrist to conduct a physical examination looking for ‘rule in’ signs such as tremor entrainment or Hoover’s sign to support the diagnosis.

Treatment begins with establishing a therapeutic alliance, psychoeducation regarding FND and its pathophysiology, and directly addressing stigma that so often limits the sufferer from accessing treatment.

Liaison with the referring neurologist, treating rehabilitation physician and allied health staff while presenting a unified front to the patient and supporting colleagues is a further central role for the psychiatrist.

Psychotherapy should be a common offering. This should be on a supportive-expressive continuum, dependent on the clinical issues at play. Indications for the continuing provision of psychotherapy are less clear. Factors such as: patient motivation, psychological mindedness and alexithymia, dissociation and attention regulation; along with the duration of symptoms and co-morbidities should all be considered in the assessment for psychotherapy process.

Beyond their use in the treatment of psychiatric comorbidities, the appropriate prescription of psychotropic medication is similarly less clear, and more evidence is needed. The judicious use of Selective Serotonin Reuptake Inhibitors in time limited trials may be justified to lower arousal and when psychotherapy alone has limited success. Patients need to be informed of the empirical nature of such a trial. The use of tricyclic antidepressants, benzodiazepines, mood stabilizers, antipsychotics, lithium and stimulants is generally reserved for patients with comorbidities where they are indicated, with the prescription choice carefully reflecting the symptom profile of the patient.

The role of the social worker

Recommendations	
C	Social workers can provide transferrable practice skills which can be utilized to promote better health outcomes for FND clients and their supporting networks.

Is there a role for a social worker in FND?

There appears to be a reluctance to diagnose people with FND, as it is an issue that almost intersects the border of neurology and psychiatry. A large gap has been identified between the treatment of people with FND versus people with organic disabilities (MS, Parkinson's, Epilepsy etc.), despite FND presenting similar symptoms. Most people living with FND and many of their carers suffer a significant decline in their mental health in correlation with living with or caring for FND symptoms[50].

It can be surmised that transferrable social work practice skills can be utilized to promote better health outcomes for FND clients and their supporting networks from a social work perspective.

What are the different roles for a social worker in FND?

The role of the social worker in FND encompasses multiple areas:

- Advocacy
- Psychosocial assessment and care planning
- Therapeutic/support intervention & adjustment/validation
- Discharge planning & community services

Advocacy

Due to the discrepancy between the treatment of FND clients when compared to the treatment of other traceable neurological issues, there is an important role for social workers to advocate for better treatment outcomes for clients in terms of accessibility to resources for assistance, as well as fair and equitable treatment by health professionals. The goal of advocacy in this field is to empower clients and their supporting partnerships with the right skills and knowledge to be able to make their own informed decisions about care and support needs.

Advocacy includes:

- Information and education for students, treating therapists, service organizations etc.
- Co-ordination of and client advocacy in family meetings
- Client advocacy in case conferencing for complex presentations
- Advocacy for appropriate services in community and for discharge planning
- Advocacy for adequate supports including disability support and access to the NDIS

Psychosocial assessment and care planning

As psychosocial assessments are a core element of practice due to their ability to gain a more complex understanding of each clients' individual situations, they are highly recommended to be utilized in this field of practice.

Psychosocial assessments and care planning in a rehabilitation context include:

- Assessment of clients' current understanding and acceptance of diagnosis, personal strengths, and effective support systems.
- Client-focused goal setting
- Identifying barriers to access therapy/achieving goals
- Therapeutic Interventions within MDT setting
- Linking to appropriate support and services

Therapeutic/support intervention & adjustment/validation

It is common for FND clients not to have a good understanding of their diagnosis initially and be unaware of the treatment/resources available. There may have been prolonged periods of symptoms with no diagnosis or misdiagnosis. Barriers to adjustment or therapeutic benefit for clients are unclear diagnosis and non-acceptance of diagnosis.

It is important for both the client and carer to understand FND, how it affects each person, and to develop effective self-care strategies- as negative mental health factors can cause significant duress if not addressed. The concept of recovery can be viewed as an overarching philosophy of practice and service delivery, which affirms the necessity of therapeutic intervention due to its use as an assistance tool for developing preventive strategies for positive mental health outcomes.

Intervention strategies may include:

- Adjustment counselling
- ACT therapy
- Emotional wellbeing management for self-care
- Anxiety and depression or grief and loss therapy

- Self-management strategies (i.e. mindfulness, locus of control, resilience etc.)
- Group education sessions

Adjustment counselling incorporating validation of FND as a diagnosable and manageable condition increases client's emotional well-being.

Benefits of feeling validated/adjustment include:

- Feeling accepted
- Feeling valued
- Generate better emotional regulation
- Strengthening of relationships.

Discharge planning/community services recommendations include:

- Discharge planning around continuity of care
- Provision of information and education for continued care, support and access to services
- Referrals to appropriate community support services.

The role of the nurse

What's the role of a nurse with an inpatient with FND?

Nurses are involved in the round-the-clock care of patients with FND. They can assist treating clinicians carry out multidisciplinary care plans (i.e. encourage physiotherapy exercises or psychology grounding exercises) and troubleshoot any issues which arise when their treating team is unavailable. Nurses can have a powerful role with helping the patient identify what makes their symptoms better and worse and document overall progress during inpatient stays by monitoring these symptoms and flares. They can also help support patients' biopsychosocial needs and improve the health literacy of patients and their families.

Uniquely to dissociative attacks, it is important that treating medical team have updated MET call criteria for their patient as appropriate. If no parameters have been set, nursing should seek clarification from the treating team. They may also need to work closely with patients and their families to educate them on the management of dissociative attacks and to find a sustainable inpatient management plan (this may include the use of midazolam in severe cases).

What is the role of a nurse (navigator) in the community for patients with FND?

Outpatient and community nurses are well positioned to help patients with FND navigate and coordinate some of the complex health care systems. They can help patients access services and act as a contact point for any issues which may arise during outpatient therapy. They can provide support within a patient's home environments such as further education, case management, social and emotional support, and clinical expertise. Community nurses many also need to manage care with the available resources available to patients in the community. This can include assisting and directing patients to access Centrelink, NDIS and GP management plans to gain access to further therapy.

The role of the rehabilitation physician

What are the key aspects of the role and scope of a rehabilitation physician?

Rehabilitation physicians have roles as doctors, advisors, educators and managers[51]. They diagnose and assess a person's function associated with injury, illness or chronic conditions, to maximise their independence and improve and maintain quality of life. They provide specialist knowledge and expertise in the prevention, assessment, management and medical supervision of a person with a disability. Rehabilitation physicians evaluate medical, social, emotional, work and recreational aspects of function. Rehabilitation physicians work with patients using an evidence based collaborative approach with other disciplines, having a unique overview of the skills and expertise of other health professionals, to develop a patient-centred, individualised treatment plan in a range of settings including home, public and private hospitals, and community rehabilitation centres and clinics[51].

What is the role of a rehabilitation physician in the diagnosis and management of FND?

Rehabilitation physicians have an important role in management patients with FND, particularly in areas where there are barriers in accessing timely neurology assessment. Rehabilitation physicians are often referred FND patients to manage as either inpatients or outpatients. Sometimes the diagnosis of FND is not clearly stated, sometimes the diagnosis has only been communicated on the referral "between the lines" and sometimes the diagnosis hadn't been made until the patient arrives in the clinic room/ward[52]. Consequently, it is often up to rehabilitation physicians to make the diagnosis, advocate for appropriate communication of the diagnosis to the patient or at least raise the possible diagnosis of FND in everyday clinical practice. Other important aspects of their role are to:

- explain the diagnosis
- validate patient experiences
- provide education
- define overarching multidisciplinary treatment plans
- manage co-existing medical concerns, including pain
- monitor progress

A rehabilitation physician's role is to collaborate with the patient to develop realistic goals for rehabilitation based on level of care required, patient's functional impairment and medical need and ability to participate in rehabilitation therapy. Goal setting can differ from familiar methods in neurorehabilitation settings where predicting recovery is usually more defined. It is recommended that a flexible approach to goal setting is required where goals are set by the patient with FND, in their own words and may not necessarily be time dependent. It is important to consider that rehabilitation will often follow a pattern of symptom remission and exacerbation[44].

Rehabilitation physicians must openly manage patients' and the treating team's expectations within a time limited inpatient admission to achieve best outcomes. The focus of management uses the biopsychosocial framework focused on improving activity limitation and participation restriction in an enriched environment that promotes effective communication, coordination of care, support, and teamwork. Ideally, rehabilitation

physicians lead a multidisciplinary team and coordinate care by facilitating regular case conferences (team meetings) and collaborate with neurologists and psychiatrists to ensure safe discharge from rehabilitation services. Several studies in inpatient and outpatient settings have shown a benefit from rehabilitation for most patients showing at least a short-term functional benefit that persists for the longer-term in many cases[53] .

For guidance on considerations for the suitability of an inpatient rehabilitation episode of care, please refer to section 'How to determine when and which sub-acute rehabilitation service is required?'.

Multidisciplinary management of FND

When is it appropriate to refer to allied health?

Not all patients with FND will require additional multidisciplinary intervention as a proportion will experience spontaneous remission. However, many patients require allied health input to assist in maximising function. Patients:

- should have an unambiguous diagnosis of FND by a physician and have this diagnosis explained to them and their key support person(s) if appropriate
- need to be at least partially receptive to the diagnosis
- need to be able to identify treatment goals and desire improvement

Patients who do not fulfil all criteria may still benefit from therapy, for example, to help them understand the basis for the diagnosis and/or assist in identifying appropriate treatment goals. Early referral of appropriate patients to appropriate disciplines is recommended given the correlation of duration of symptoms with poorer outcomes.

When is it appropriate to trial and use assistive devices?

The use of aids, equipment, splints, adaptation, and long-term modifications to environment are generally not recommended as part of the rehabilitation process in FND, particularly in the acute phase. This is because they may encourage maladaptive movements patterns and behaviours and potentially lead to reliance and further deconditioning and/or pain.

There are times when providing equipment or modifications are necessary and appropriate. In these situations (i.e., for safe discharge from hospital) they should be:

(1) considered as a short-term solution, with a plan to progress from it use; and

(2) issued with a minimalist approach; and

It is important to assess the person with new equipment and teach them how to use it correctly to minimise maladaptive movement patterns. Follow-up appointments should be available to monitor equipment use and support plans to progress towards independence.

Equipment or modifications may be appropriate if a person has completed rehabilitation, has treatment resistant symptoms and/or is living with a potentially long-term/chronic disability. In this case, equipment and modifications may be required to improve safety, optimise a person's quality of life and support engagement in activities of daily living[44].

I have been referred a patient without a definitive diagnosis of FND / I suspect my patient has FND.

Having a confirmed diagnosis of FND and having this diagnosis explained to the patient are important steps in successful management. Having said that, in regular clinical practice the diagnosis is often missed and/or not communicated by the treating doctors, which can make treating patients with FND challenging [16,52]. This is clearly not an ideal situation, but there are still things all health professionals can do to help.

General approach

Observe and share target symptoms, including:

- Fluctuations/discrepancies
- Triggers
- Duration
- Time of day phenomena
- Communicate observed positive signs - the inconsistencies and incongruities between impairment testing and function. Comment on the impact of aggravating and easing factors reflective of the biopsychosocial model.

Communicate the impact on management when diagnosis is unknown to patients. This may include ongoing abnormal health beliefs (i.e. patients worried they have a neurodegenerative disease or wanting further investigations) or anxiety of not understanding perpetuating factors on symptoms (i.e. the role of psychosocial factors on functional gait disturbance).

These factors limit the effect of treatment interventions and the level of acceptance and engagement in therapies by the patient.

Inpatient setting

- Take note of your observations and positive signs of FND
- Communicate with other team members (for example in case of motor symptoms discuss with physiotherapist or occupational therapist to assess further for positive FND signs)
- Discussion with the broader medical and multidisciplinary team to consider further assessment by medical specialists
- Even in the absence of a positive diagnosis of FND and if guided by the medical team, it can be appropriate to use an FND treatment approach

Community setting

- Discussion with multidisciplinary team if possible and referral to other allied health if able for review i.e., physiotherapy for functional mobility/pain assessment, positive FND signs, social worker for psychosocial assessment, speech pathologist if speech, swallow, voice involved.
- Discussion with patient's GP, recommended via phone call and follow up with written recommendation for referral to neurologist to assess for possibility of FND as the cause for symptoms
- While awaiting formal assessment, FND treatment approaches if appropriate, ensuring a no harm approach

Ensure there is ongoing allied health follow up as appropriate to your service and region.

Lehn A, *et al. BMJ Neurol Open* 2025; 7:e000970. doi: 10.1136/bmjno-2024-000970

How will my team handle complexity?

Complex presentations can be scary and daunting for any health professional and the truth is that outcomes in FND are often poor in these cases[42]. However, for the vast majority of FND patients we can help in some way. A good explanation can go a long way for most patients and even just showing patients that they are heard and being taken seriously is very helpful[32]. We all can add to a patient's treatment, even if we are not specialists in this field. This should include good communication with patients, their families and other health professionals. The observation and sharing of target symptoms in a non-judgmental way can be helpful in management of symptoms. Good education about underlying mechanisms leading to functional symptoms and treatment principles is important. It can be empowering for junior team members and other involved health professionals with less experience to know that they can have an important role to play in patient management. Poor attitudes towards patients with FND are still common among health professionals and they are often brought on by lack of education and poor support[24]. An improved knowledgebase, better management approaches and more positive attitude can be taught with relatively little resources[54]. A good team approach to complex patients is important, where we not only support the patient but also support the other team members. Regular meetings by the treating team to discuss current issues and future care planning can be helpful.

General advice regarding complex cases

Not every FND patient needs to see a psychiatrist, but in certain situations it can be very helpful (for example in the diagnosis and treatment of co-existing severe anxiety and depression). We are fortunate in Queensland to have several excellent (neuro)psychiatrists in public and private clinics. If you struggle in connecting your patient with a psychiatrist you can find a list of people with interest in FND on the website fndaustralia.com.au or you can contact the FND special interest group on FND.SIG@health.qld.gov.au.

For functional movement disorders it is often helpful to get a good physiotherapist involved who has experience in treatment of FND and for dissociative attacks a good psychologist with expertise in this area. It can be very helpful if patients are seen by a psychologist AND physiotherapist. This can be difficult to organise if you don't have the experience in your team, but you may like to link in with more experienced therapists (for example members of the FND SIG) for support and supervision. This can not only help you with management of your patient but also gives the opportunity to train the team further and build confidence in managing more complex patients in the future.

Are there services available for patients with severe and intractable functional symptoms?

These patients can be particularly challenging, and our current health system is not well set up to manage them. There are some services available through the public system (like chronic pain clinics) but there is a need for high-level multidisciplinary services in this area. Hopefully this is something that will be developed through the public system in Queensland at some point in the future. Access to the NDIS can help these patients to set up appropriate therapy and support structures.

When is the right time to discharge a patient from multidisciplinary services?

It is important to recognise that FND can be a chronic condition, even after multidisciplinary intervention. Consequently, emphasis on self-management is vital from initial contact to

formulate a follow up plan and a clear discharge summary. Relapses frequently occur and people with FND need to be taught the skills to self-manage them. Considerations for discharge:

- Service limitations: from the onset, be clear about what the available resources are (i.e. are there session or time limitations? Is your role rotational or will you be consistently managing from your discipline?)
- Communication: ensure the patient understands the link between their goals and the service goals (i.e. is it realistic for them to achieve their goal within your service, or will ongoing self-management be required?)
- Goals: has there been a limit to what a service can provide a patient at this stage of their recovery?
- Biopsychosocial Model: have all perpetuating factors been managed within the current service?
- Follow up plan: who will continue to manage the patient once they discharge from your service? Who can they contact if their function regresses? Has a handover been provided or your contact details shared?
- Self-management: has the service developed these skills with the patient? Use of an FND self-management booklet is an appropriate tool to ensure the patient has the relevant self-management information provided to them (see Resources).

It is recommended to provide appropriate handover (e.g in the form of a discharge summary) to the referrer, general practitioner and the patient themselves. Note that FND can co-exist with other pathologies, so if new symptoms emerge, these need to be clinically assessed.

Return to work/school

Returning to work or school can be a trigger for some people with FND. This goal may require a graded approach with input from a multidisciplinary rehabilitation team in consultation with the patient and appropriate professionals within the work and/or school environment. Return to work principles for neurological rehabilitation can be applied to people with FND. With permission of the patient, return to work may include education to employers or school staff regarding symptoms of FND and that it can have periods of remission and exacerbation. The patient may require professional support to implement a graded return to work or school plan. This may include advice regarding environmental and/or role modifications to help manage symptoms including scheduled rest breaks and reduced hours/days. This initial plan may then be progressed with ongoing input of the multidisciplinary team.

Access to disability support and navigating the NDIS

In some cases, sustaining paid employment may not be possible and support to seek alternative roles may be required.

Eligibility for NDIS

Before the NDIS existed, there was little support available for people with disability under the age of 65. . The problem is that FND by its very nature is variable and prognosis may be unclear. Decisions about support via the NDIS are made by people who do not have medical training and who usually only have limited knowledge of FND. Common obstacles when trying to access NDIS are questions of permanency and therapies tried in the past. Unless a patient's FND is considered a permanent condition and every feasible treatment has been attempted prior to applying, there is generally little chance of a successful application. In

most cases a successful application requires specialist reviews to provide supporting information. Additionally, external advice when trying to access the scheme may be helpful. This can be through freely available support like Carers Queensland (carersqld.com.au) or through private support agencies (which usually involves extra costs).

FND is currently not a listed condition that gives automatic approval under NDIS. However, depending on other coexisting diagnoses, funding can sometimes be accessed if the individual's condition is deemed to be chronic with minimal improvement expected. This should be discussed with the individual's neurologist and other members of the MDT if possible.

Often members of the multidisciplinary team are asked to provide supporting documentation for the patient to complete the Access Request Form. Some tips:

- Commenting on what the patient can't do physically compared to what they can do. For example, "Patient is unable to walk independently and requires the assistance of one and a mobility aid to mobilise indoors and outdoors. Without these, the patient is at risk of falls and harm, and thus functional deterioration."
- Using objective measures to capture symptoms not improving over time including physical measures (Simplified Functional Movement Disorder Rating Scale, Ten Metre Walk Test, Patient Specific Functional Scale) and subjective measures (The Modified Fatigue Impact Scale, The Brief Illness Perception Questionnaire, Short Form 36 Health Survey Questionnaire). Another helpful tool is the WHO Disability Assessment Schedule (WHODAS 2.0) as this covers the domains relevant to NDIS – Self-Care, Mobility, Self-Management, Community Participation, Social Wellbeing, Learning and Communication.
- The NDIS does not fund rehabilitation, but rather maintaining function to build capacity and optimise participation in day-to-day life. It is important to collaborate with the patient to establish broad overall participation goals at their current level of function.

FND in elderly patients

Recommendations	
C	FND in elderly is common, however diagnosis can be more challenging due to the frequency of comorbidities.
✅	Better education would raise awareness of FND amongst General Practitioners and Geriatricians and thus its identification in clinical practice.

Can elderly people have FND and how common are FND symptoms in the elderly patient?

Functional Neurological Disorders in the elderly are not uncommon. For example, in a study specific to neurogeriatric inpatients, the prevalence of functional movement disorders after the age of 60 was reported to be as high as 20%[55]. They also found a statistically significantly greater prevalence of functional movement disorder in geriatric females over males. There appears to be a particular link between functional disorders and Lewy body pathology: The rates of FND and somatic symptom disorders are higher in Parkinson's Disease and dementia with Lewy bodies than in patients with Alzheimer's dementia or atypical parkinsonism[56–58].

Lehn A, et al. BMJ Neurol Open 2025; 7:e000970. doi: 10.1136/bmjno-2024-000970

What are the most common presentations of FND in the elderly?

Dissociative attacks are a common functional symptom seen in the elderly and are classified as a subtype of FND in the DSM-5. It is estimated that up to 30% of admissions to epilepsy monitoring units are diagnosed with dissociative attacks on discharge. Dissociative attacks are equally distributed across the sexes in a geriatric population[13]. Presentation of a distractable tremor in upper limbs or another part of the body e.g. truncal is another common functional neurological symptom that is found in the elderly[55].

What are the red flags or clues to look for in considering FND in the elderly?

Various aging-associated factors, such as functional decline, psychosocial problems, and cognitive dysfunction, are risk factors for somatoform disorders (SDs) in the elderly. Depression correlates with anxiety and somatic symptoms in the elderly. Consideration to emotional stability, dominance and vigilance as well as educational status have been found to influence propensity for somatization.

How to diagnose and treat FND in the elderly patient?

The principles of diagnosis and general treatment approaches of FND in the elderly are the same as in other age groups. The diagnosis may be more challenging in geriatric versus non-geriatric patients given the comorbidities, inclusive of additional neurodegenerative disorders, in this population.

FND in Aboriginal and Torres Strait Islander people

Recommendations	
✅	Patients' cultural backgrounds should be considered in management, with good communication being a cornerstone of management.
✅	The 'yarning' approach is a socially acceptable method of communication in an Aboriginal context.

How do you include the patient's background in the cross-cultural treatment situation, particularly considering Aboriginal and Torres Strait Islander people?

A few considerations for functional neurological disorder in Aboriginal and Torres Strait Islander people are important. For example, protective and risk factors must be considered in the broadest possible sense, including social determinants of health, local community and culture and the impact of current and historical systems on trust. Also, expectations need to be managed accordingly in this group of patients. FND can be a highly conceptual and challenging concept. Management often involves re-framing symptoms to create a healing rather than damaging outlook. Given the many barriers that exist between Indigenous and non-Indigenous people, good communication is a cornerstone of management.

Lehn A, *et al. BMJ Neurol Open* 2025; 7:e000970. doi: 10.1136/bmjno-2024-000970

What are helpful considerations when communicating with Aboriginal and Torres Strait Islander people?

The "yarning" approach in consultations is a socially accepted way of communicating in an Aboriginal context[59]. There are parallels with bilateral story-telling, where listening and reflecting are important components of the communication process. There is emphasis on getting to know the person and then putting the issue in this context. The person's behaviours and aspirations are informed by the surrounding environment (e.g. family, community), so these must be appreciated. The yarning approach requires an atmosphere of trust and time. There is decreased reliance on clinical measurement tools, due to the highly individualised nature of the approach.

It is important to understand that the yarning approach is not simply a clinical skill. The system that is used to deliver care must be optimised to facilitate yarning. Barriers to access (e.g. physical, social, cultural, financial, geographic) need to be actively addressed to create a trusting and accessible environment. The clinician needs to be comfortable with reducing the natural power differential which exists in the patient-clinician relationship. Clinician humility, and even vulnerability, is necessary to deeply understand the patient's needs.

When considering the influence of culture and context, there is no neat answer. The solutions must be found via the clinician's genuine curiosity and willingness to help, which will hopefully unveil the specific cultural and contextual factors for the individual in front of you.

Health care systems in FND

Recommendations	
✅	The use of subacute rehabilitation services to manage patients with FND should be considered on a case-by-case basis.
**B**	The use of telehealth as delivery mode for Cognitive Behavioural Therapy (CBT) is helpful in managing dissociative events.
**C**	The use of telehealth to delivery physiotherapy and psychiatry to promote self-management and for movement retraining.

Is a doctor needed as part of the FND treatment team?

A doctor is not necessarily needed as part of the FND treatment team, particularly in milder cases with a clear diagnosis. Doctors have an important role though in making the diagnosis of FND and in the communication of this diagnosis to the patient, the family and other health professionals[60]. The current evidence base supports a multidisciplinary approach to the treatment of functional neurological symptoms[7,45]. As part of a multidisciplinary team doctors have a role to help coordinate management. Also, in complex patients (for example where there is a coexistence of organic and functional processes or when new symptoms emerge) it is helpful to have a doctor in the team to reassess and/or investigate a patient if necessary.

What should be done if a patient with FND presents acutely to hospital?

Education for all health professionals involved in the management of acute FND is important and can have a major impact on improving diagnosis and management[61,62]. This includes paramedics, triage nurses and emergency department staff.

FND patients may also come into inpatient settings with a history of clinical disbelief and trauma from experience with the medical system. All staff in the ED departments, but particularly the attending consultant and their registrar, have a significant role to play not only in assessing physical symptoms but in assuring the patient that they believe what they are saying about their illness and lived experience thus allaying their fears, and setting up a positive environment to explain their diagnosis. If acute presentation at ED is the first presentation a person has with their physical FND symptoms they may be very *worried* they are having a stroke or another organic disease. A positive and sensitive clinical reaction to this situation can be life-changing for an FND patient, helping them bypass the stigma that others have endured, and giving them the best chance of improvement early.

Successful management of acute FND presentations in ED can be very helpful in the short term but can also have a positive therapeutic effect in the long term. Some patients may be discharged home directly from ED or after a short-stay in a medical assessment or short-stay unit. Some cases may need to be admitted for care as an inpatient for further assessment; or they may be admitted to a mental health ward if experiencing a mental health crisis. Successful treatment as an inpatient warrants a well organised team approach, but in the right setting and for the right patient can be very successful[63].

Access to dedicated neurology wards and multidisciplinary teams is limited in Queensland hospitals[52] Whilst being admitted as an inpatient can be an appropriate course of action, FND patients can also find it challenging to be in a shared ward setting where they involuntarily become part of other patients' trauma, conversations, and challenges. In the public system, it is rare for FND patients to be able to have their own room or even to have a 'quiet' space where they can go to debrief and re-calibrate. Further, other patients and their families, or staff may not appreciate the more hidden aspects of FND e.g. sensory overload, cognitive challenges, or associated anxiety, and judge the FND patient insensitively based on what they can only physically see. FND patients may then believe what is said to them or insinuated is that they are not as sick as other inpatients and then they may not seek help when they should speak out. Clinicians need to be mindful of these situations and ensure that FND patients receive the best care possible.

The transition to community services or at home rehabilitation can also be challenging to organise when services are limited and education about FND is poor amongst the outpatient staff. Availability in rehabilitation centres is limited for FND patients and hospitals have their own rules about taking FND patients into their outpatient facilities e.g. use of the outpatient physiotherapy services in an onsite gym.

What are the models of care for therapy of FND and how can they be accessed in Queensland?

There are several inpatient and outpatient models of care used in the therapy of FND, which can be used in different types of patients with FND according to their needs. Published reports of inpatient and outpatient programs have varying treatment durations, multidisciplinary approaches and intensity. For excellent summaries of the current literature

we recommend: Physiotherapy for functional motor disorders: a consenus recommendation[30], Occupational therapy consensus recommendations for functional neurological disorder[44] or Treatment of Functional Movement Disorders[45]. General rehabilitation themes include: demonstrating that normal movement can occur, retraining normal movement, addressing secondary changes and providing education.

What is available to help with the hospital to community transition on discharge?

Individuals with FND would likely benefit from referral to community services for immediate follow up following a hospital admission to support transition home and assess triggers/function in the home, work and community environments and optimise self-management and reduce the risk of readmission.

When discharging a patient who has functional neurological symptoms it is important to consider what formal and informal support they may have available and provide referrals as clinically appropriate. Most public hospital and health services have a domiciliary service including Rehabilitation In The Home (RITH) and hospital outpatient services to support transition home and return to usual activities. The accessibility to public services varies largely across the state and is less likely to be available in regional and remote areas.

Current Queensland Health specialised FND programs

Horizons Program – Gold Coast University Hospital

The Horizons Team at the Gold Coast University Hospital provides an outpatient clinic once a week to children and adolescents up to 18 years with FND. It is a multi-disciplinary team involving a paediatrician, psychologist, physiotherapist and occupational therapist and more recently psychiatric consultancy. Only people within the Gold Coast Health service catchment are eligible.

FND Education Program – Cairns Base Hospital

This is an outpatient education program that runs face to face or virtually via telehealth. It is delivered once a week over four weeks. The team accepts referrals directly to Cairns Adult Community Health via the centralized intake – chhhs-centralreferralintake@health.qld.gov.au. Referrals are accepted from any doctor or allied health clinician provided there is an established FND diagnosis. Inclusion criteria is that the patient must have been provided with a definitive diagnosis of Functional Neurological Disorder by a neurologist and reside within the Cairns and Hinterland Health Service District. Topics covered include 'What is FND', 'Optimizing resilience', 'Identifying and managing triggers', 'Managing fatigue and memory strategies' and the program is delivered by a physiotherapist and occupational therapist. Participants may also have a concurrent referral to a relevant health professional at community health (e.g. physiotherapy for movement issues, speech pathology for speech issues).

FND Clinic – Sunshine Coast University Hospital

The Sunshine Coast FND Treatment Clinic offers a six-session integrated multitdisciplinary intervention with joint sessions by a physiotherapist, clinical psychologist and neurologist. The goals of treatment are to induce remission or functional recovery in this time and teach strategies for self-management of ongoing symptoms and possible future relapses. Referrals are accepted by e-blue slip from within the SCHHS catchment for anyone with FND who has seen a neurologist within the last year. People who have seen a neurologist in the past need

confirmation by a neurologist that their current symptoms are still attributable to FND before they are referred.

Private FND services in Queensland

Various private multidisciplinary services exist in the community and through private day hospital models. A directory of health professionals can be found on the FND Australia website (fndaustralia.com.au). Support for people living in the community with functional symptoms is often limited to what can be paid for privately, or what might be funded under NDIS or MAC. Patients with functional neurological symptoms can access a Mental Health Treatment Plan via their GP, which assists with accessing up to 20 Medicare-subsidised psychology sessions per calendar year. They may also be able to access an Enhanced Primary Care Plan via their GP, allowing for up to 5 Medicare-subsidised allied health sessions per calendar year.

How to determine when and which sub-acute rehabilitation service is required?

In ideal situations, acute presentations of FND should progress to a functionally safe level for discharge on the acute ward and then be linked with community services for ongoing management. Unfortunately, the complexity of FND presentations in hospital varies significantly and intensive management may be required over a longer period. The options available include intensive therapy on the acute ward or admission to an inpatient rehabilitation unit.

All patients referred for rehabilitation should be assessed and considered case-by-case basis (rather than an “all included” or “all excluded” criteria). Considerations when determining when and which sub-acute inpatient services to refer to:

- ***Perpetuating factors:*** are there perpetuating factors that favour other available specialist service involvement including Mental Health or Persistent Pain management prior to or post inpatient rehabilitation?
- ***Sub-acute resource availability:*** whether skilled and knowledgeable clinicians lead the service, bed capacity, waiting lists and environmental concerns.
- ***Patient factors:*** poor functional progression on the acute ward, poor acceptance of the diagnosis, inability to identify therapy goals, reduced participation in the full multidisciplinary approach and presence of dissociative events are poor prognostic factors for linear recovery.
- ***Functional cognitive symptoms*** in isolation are less responsive to therapeutic approaches. Alternative compensatory strategies may need to be explored (e.g., external assistance from formal and informal supports via NDIS, services, family and friends).
- Goals should be mutually agreed upon between all stakeholders, with clear communication if the goals change. For example, when there is a need to move towards compensatory strategies to enable discharge.
- ***Team consistency:*** communication is pivotal not only with managing the patient clinically, but also to mitigate the risk of the patient decompensating functionally over time. This can be due to over-reliance on the health care service, persistent anxieties around safe transition home and inconsistencies of information delivered by the team, particularly during team rotation changes.

How can I manage FND with limited resources in regional and remote communities?

The management of FND in regional and rural communities is particularly difficult due to the lack of resources outside of metropolitan areas. The situation in Queensland is particularly challenging, with vast distances separating health care settings, with a significant divide between “Metro-centric” Southeast Queensland, and the rest of the state. The prognosis/outcomes of FND patients in these settings is not well studied, although the outcomes for most patients with chronic illnesses tend to be poorer in these locations for various reasons. Clinicians from regional and remote locations are likely to be less confident in treating patients with FND due to reduced training and support from skilled clinicians and have far fewer resources in order to do so. This is pertinent at all levels of a patient’s recovery, including on the acute ward, inpatient rehabilitation and ambulatory rehabilitation services. With high staff turnover in these locations due to difficulties with attraction and retention, skilled clinicians may leave these services, leaving entry-level staff without guidance in appropriate treatment strategies and education for these patients. Moreover, regional and rural locations generally lack the infrastructure to manage these patients at all levels, with reduced inpatient bed capacity both in acute and rehabilitation units, and difficulty for patients to access their nearest ambulatory rehabilitation centre due to legal driving ramifications, distance and financial cost.

Despite all these challenging issues (which are not unique to FND) it is still possible to help patients in low resource settings. The first step of treatment is diagnosis and a good explanation, which could be provided by a doctor with some training in this area (perhaps with support and/or supervision by a specialist). Alternatively, the patient could initially be referred to a specialist for diagnosis and then managed again locally. For milder cases, targeted therapy could be provided by a local therapist or multi-disciplinary team with adequate support by specialist services. Increasing communication and supports from more metro-centric Queensland locations will assist clinicians in regional and remote locations to provide high quality, effective, patient-centred subacute services for our FND patients. Moreover, there needs to be recognition that this vulnerable patient group is generally under recognised, underfunded and thus, poorly managed throughout Queensland; the result of this to date, is excessive strain on those very few designed services and skilled clinicians, and significant morbidity in our patients. Telehealth services are increasingly used in order to bridge the divide between patients and service providers. Telehealth has the benefit of reducing the impact on location, transport time/costs and a lack of local skilled clinicians on the provision of medical services. Whilst it does not replace face-to-face reviews, it may link more isolated patients with providers to enable appropriate treatment. Specific to FND telehealth can been useful for:

Cognitive Behavioural Therapy (CBT)[64]. CBT can incorporate workbooks, technology applications (“apps”) and traditional therapist-led CBT.

Physiotherapy and psychiatry promoting long term self-management strategies and movement retraining[65].

Governmental (and nongovernmental) bodies need to increase funding to develop and foster appropriate resources and upskilling of clinicians to assist these patients in their rehabilitation management.

FND co-existing with persistent pain

Is there a clinical overlap between persistent pain and FND?

Persistent pain may be a condition of the central nervous system where the normal physiological response of pain becomes overprotective or sensitised and there is an inability of the CNS to adequately downregulate its protective function[66]. Similar to FND, this process is mediated by biopsychosocial predisposing, triggering and perpetuating factors. Due to the complexity of FND and persistent pain, they should be approached with a rehabilitation and management focus rather than curative[67].

Current evidence is supportive of a using the biopsychosocial framework as the most efficacious approach to the management of both FND and persistent pain[63,68]. This allows consideration of all factors that may be contributing to the patient's overall symptoms and disability.

When should I refer patients to neurology/rehabilitation services vs a persistent pain clinic?

Severe persistent pain is a poor prognostic factor for people with FND and is often the most significant perpetuating factor causing disability and altered mood. Due to this, addressing pain and associated mood changes first may be appropriate. However, as specialty services are scarce in Queensland, there are a variety of factors to consider:

- After completing a thorough examination, determine which of the perpetuating factors are most impacting functional symptoms, the pain profile and disability. Referring to the biopsychosocial model (table 1) will assist with this.
- Waitlists to local public pain services are very long in much of Queensland. It is imperative that patients remain linked (this could mean spacing out patient review cycles) to a service whilst they are waiting to avoid the negative effects of symptom chronicity without management.
- Multidisciplinary approach is best for complex cases in both instances, either speciality service will be able to provide some guidance to the patient where services aren't available.

Where do I start when treating patients with FND with co-existing persistent pain?

Similarities in the presentation of FND and co-existing persistent pain allow the clinician a unique opportunity to address commonalities simultaneously. Research indicates that a multidisciplinary team approach may be of benefit for persistent pain management. Furthermore, persistent pain may be better managed when interventions are combined, such as exercise and education, rather than sole treatment approaches like exercise alone[68,69]. Common overlaps in management of of FND and persistent pain include:

- Appropriate diagnosis, explanation/patient understanding of persistent pain (and FND) using a BSP framework[66].
- Advice and education addressing the nervous system, contextual factors, beliefs and misconceptions, expectations, stress and psychological factors, pacing and graded return to activity/exercise and flare up management[66].

- Sleep[70]
- Strategy development including psychological and physical strategies to reduce the impact of triggers on pain and activity engagement[66,67]
- Psychological support and building resilience[70].

References

1. Schmidt, T. *et al.* Evaluation of Individualized Multi-Disciplinary Inpatient Treatment for Functional Movement Disorders. *Mov Disord Clin Pract* (2021) doi:10.1002/mdc3.13268.

2. Nielsen, G. *et al.* Randomised feasibility study of physiotherapy for patients with functional motor symptoms. *Journal of neurology, neurosurgery, and psychiatry* jnnp-2016-314408 (2016) doi:10.1136/jnnp-2016-314408.

3. Goldstein, L. H. *et al.* Cognitive behavioural therapy for adults with dissociative seizures (CODES): a pragmatic, multicentre, randomised controlled trial. *Lancet Psychiatry* **7**, 491–505 (2020).

4. Scotland, H. I. *Stepped Care for Functional Neurological Symptoms*. (2012).

5. Rosenfeld, R. M. & Shiffman, R. N. Clinical Practice Guideline Development Manual: A Quality-Driven Approach for Translating Evidence into Action. *Otolaryngol.Head Neck Surg.* **140**, S1–S43 (2009).

6. Rosendal, M., Christensen, K. S., Agersnap, L., Fink, P. & Nielsen, C. V. *Functional Disorders*. (Birgitte Dansgaard, Komiteen for Sundhedsoplysning, 2013).

7. Aybek, S. & Perez, D. L. Diagnosis and management of functional neurological disorder. *Bmj* **376**, o64 (2022).

8. Espay, A. J. *et al.* Current Concepts in Diagnosis and Treatment of Functional Neurological Disorders. *JAMA neurology* (2018) doi:10.1001/jamaneurol.2018.1264.

9. Liotti, G. A Model of Dissociation Based on Attachment Theory and Research. *J. Trauma Dissociation* **7**, 55–73 (2006).

10. Guérin-Marion, C., Sezlik, S. & Bureau, J.-F. Developmental and attachment-based perspectives on dissociation: beyond the effects of maltreatment. *Eur. J. Psychotraumatology* **11**, 1802908 (2020).

11. Ludwig, L. *et al.* Stressful life events and maltreatment in conversion (functional neurological) disorder: systematic review and meta-analysis of case-control studies. *The Lancet Psychiatry* **5**, 307–320 (2018).

12. Stone, J. *et al.* Symptoms “unexplained by organic disease” in 1144 new neurology out-patients: how often does the diagnosis change at follow-up? *Brain : a journal of neurology* **132**, 2878–2888 (2009).

13. Carson, A. & Lehn, A. Epidemiology. *Handbook of Clinical Neurology* **139**, 47–60 (2017).

14. Stone, J. *et al.* Who is referred to neurology clinics?--the diagnoses made in 3781 new patients. *Clinical neurology and neurosurgery* **112**, 747–751 (2010).

15. Ahmad, O. & Ahmad, K. E. Functional neurological disorders in outpatient practice: An Australian cohort. *J Clin Neurosci* **28**, 93–96 (2016).

16. Lehn, A. *et al.* Psychogenic nonepileptic seizures treated as epileptic seizures in the emergency department. *Epilepsia* (2021) doi:10.1111/epi.17038.

17. Hallett, M. *et al.* Functional neurological disorder: new subtypes and shared mechanisms. *Lancet Neurology* (2022) doi:10.1016/s1474-4422(21)00422-1.

18. Butler, M. *et al.* International online survey of 1048 individuals with functional neurological disorder. *Eur. J. Neurol.* **28**, 3591–3602 (2021).

19. Carson, A. *et al.* Disability, distress and unemployment in neurology outpatients with symptoms “unexplained by organic disease”. *Journal of neurology, neurosurgery, and psychiatry* **82**, 810–813 (2011).

20. Trimble, M. & Reynolds, E. H. A brief history of hysteria: From the ancient to the modern. *Handbook of clinical neurology / edited by P.J. Vinken and G.W. Bruyn* **139**, 3–10 (2017).

21. Stone, J. *et al.* What should we say to patients with symptoms unexplained by disease? The “number needed to offend.” *BMJ* **325**, 1449–1450 (2002).

22. Lagrand, T. J., Jones, M., Bernard, A. & Lehn, A. C. Health Care Utilization in Functional Neurologic Disorders: Impact of Explaining the Diagnosis of Functional Seizures on Health Care Costs. *Neurology Clin Pract* **13**, e200111 (2023).

23. Edwards, M. J., Yogarajah, M. & Stone, J. Why functional neurological disorder is not feigning or malingering. *Nat Rev Neurol* 1–11 (2023) doi:10.1038/s41582-022-00765-z.

24. Lehn, A., Bullock-Saxton, J., Newcombe, P., Carson, A. & Stone, J. Survey of the perceptions of health practitioners regarding Functional Neurological Disorders in Australia. *J Clin Neurosci* **67**, 114–123 (2019).

25. Merckelbach, H., Dandachi-FitzGerald, B., Helvoort, D. van, Jelicic, M. & Otgaar, H. When Patients Overreport Symptoms: More Than Just Malingering. *Curr. Dir. Psychol. Sci.* **28**, 321–326 (2019).

26. Kranick, S. M. *et al.* Action-effect binding is decreased in motor conversion disorder: implications for sense of agency. *Movement Disorders* **28**, 1110–1116 (2013).

27. Pareés, I. *et al.* Loss of sensory attenuation in patients with functional (psychogenic) movement disorders. *Brain : a journal of neurology* **137**, 2916–2921 (2014).

28. Gelauff, J., Stone, J., Edwards, M. & Carson, A. The prognosis of functional (psychogenic) motor symptoms: a systematic review. *Journal of neurology, neurosurgery, and psychiatry* **85**, 220–226 (2014).

29. Durrant, J., Rickards, H. & Cavanna, A. E. Prognosis and outcome predictors in psychogenic nonepileptic seizures. *Epilepsy research and treatment* **2011**, 274736 (2011).

30. Nielsen, G. *et al.* Physiotherapy for functional motor disorders: a consensus recommendation. *Journal of neurology, neurosurgery, and psychiatry* **86**, 1113–1119 (2015).

31. Stone, J. Functional neurological disorders: the neurological assessment as treatment. *Neurophysiologie clinique = Clinical neurophysiology* **44**, 363–373 (2014).

32. Stone, J. Functional neurological disorders: the neurological assessment as treatment. *Practical neurology* **16**, 7–17 (2016).

33. Edwards, M. J. Functional neurological disorder: lighting the way to a new paradigm for medicine. *Brain* **144**, 3279–3282 (2021).

34. Stone, J. & Edwards, M. Trick or treat? Showing patients with functional (psychogenic) motor symptoms their physical signs. *Neurology* **79**, 282–284 (2012).

35. Diagnostic And Statistical Manual Of Mental Disorders, Fifth Edition, Text Revision (DSM-5-TR). (2022) doi:10.1176/appi.books.9780890425787.

36. Organization, W. H. *International Statistical Classification of Diseases and Related Health Problems*. (World Health Organization, 2021).

37. Stone, J., Burton, C. & Carson, A. Recognising and explaining functional neurological disorder. *BMJ* **371**, m3745 (2020).

38. Stone, J. *et al.* Systematic review of misdiagnosis of conversion symptoms and “hysteria”. *BMJ* **331**, 989 (2005).

39. Walzl, D., Carson, A. J. & Stone, J. The misdiagnosis of functional disorders as other neurological conditions. *J. Neurol.* **266**, 2018–2026 (2019).

40. Benbadis, S. R., Agrawal, V. & Tatum, W. O. How many patients with psychogenic nonepileptic seizures also have epilepsy? *Neurology* **57**, 915–917 (2001).

41. Tinazzi, M. *et al.* Functional motor disorders associated with other neurological diseases: Beyond the boundaries of “organic” neurology. *Eur J Neurol* **28**, 1752–1758 (2021).

42. Gelauff, J. & Stone, J. Prognosis of functional neurologic disorders. *Handbook of clinical neurology / edited by P.J. Vinken and G.W. Bruyn* **139**, 523–541 (2017).

43. Nielsen, G. Physical treatment of functional neurologic disorders. *Handbook of clinical neurology / edited by P.J. Vinken and G.W. Bruyn* **139**, 555–569 (2017).

44. Nicholson, C. *et al.* Occupational therapy consensus recommendations for functional neurological disorder. *J Neurology Neurosurg Psychiatry* **91**, 1037–1045 (2020).

45. LaFaver, K. Treatment of Functional Movement Disorders. *Neurol. Clin.* **38**, 469–480 (2020).

46. Baker, J. *et al.* Management of functional communication, swallowing, cough and related disorders: consensus recommendations for speech and language therapy. *J Neurology Neurosurg Psychiatry* jnnp-2021-326767 (2021) doi:10.1136/jnnp-2021-326767.

47. Verdonschot, R. J. C. G. *et al.* Medically Unexplained Oropharyngeal Dysphagia at the University Hospital ENT Outpatient Clinic for Dysphagia: A Cross-Sectional Cohort Study. *Dysphagia* **34**, 43–51 (2019).

48. Roth, C. R., Cornis-Pop, M. & Beach, W. A. Examination of validity in spoken language evaluations: Adult onset stuttering following mild traumatic brain injury. *NeuroRehabilitation* **36**, 415–26 (2015).

49. *Diagnostic and Statistical Manual of Mental Disorders (5th Ed.; DSM-5)*. (American Psychiatric Association, 2013).

50. *Experiences of Functional Neurological Disorder Summary Report*. (2019).

51. Medicine, A. F. of R. The Role of the Rehabilitation Physician. https://www.racp.edu.au/docs/default-source/advocacy-library/role-of-the-rehabilitation-physician.pdf (2008).

52. Petrie, D. *et al.* How Is Functional Neurological Disorder Managed in Australian Hospitals? A Multi-Site Study Conducted on Acute Inpatient and Inpatient Rehabilitation Wards. *Mov Disord Clin Pract* (2023) doi:10.1002/mdc3.13718.

53. Gilmour, G. S. & Jenkins, J. D. Inpatient Treatment of Functional Neurological Disorder: A Scoping Review. *Can J Neurological Sci J Can Des Sci Neurologiques* **48**, 204–217 (2021).

54. Lehn, A. *et al.* Functional neurological disorders: effective teaching for health professionals. *Bmj Neurology Open* **2**, e000065 (2020).

55. Batla, A. *et al.* Functional movement disorders are not uncommon in the elderly. *Movement disorders : official journal of the Movement Disorder Society* **28**, 540–543 (2013).

56. Onofrj, M., Bonanni, L., Manzoli, L. & Thomas, A. Cohort study on somatoform disorders in Parkinson disease and dementia with Lewy bodies. *Neurology* **74**, 1598–1606 (2010).

57. Onofrj, M. *et al.* Updates on Somatoform Disorders (SFMD) in Parkinson's Disease and Dementia with Lewy Bodies and discussion of phenomenology. *Journal of the neurological sciences* **310**, 166–171 (2011).

58. Onofrj, M. *et al.* Functional neurological disorder and somatic symptom disorder in Parkinson's disease. *J Neurol Sci* 120017 (2021) doi:10.1016/j.jns.2021.120017.

59. Burke, A. W., Welch, S., Power, T., Lucas, C. & Moles, R. J. Clinical yarning with Aboriginal and/or Torres Strait Islander peoples—a systematic scoping review of its use and impacts. *Syst. Rev.* **11**, 129 (2022).

60. Stone, J., Carson, A. & Hallett, M. Explanation as treatment for functional neurologic disorders. *Handbook of clinical neurology / edited by P.J. Vinken and G.W. Bruyn* **139**, 543–553 (2017).

61. Grönheit, W. *et al.* Teaching distinguishing semiological features improves diagnostic accuracy of seizure-like events by emergency physicians. *Neurological Res Pract* **4**, 56 (2022).

62. Williams, S. *et al.* To the emergency room and back again: Circular healthcare pathways for acute functional neurological disorders. *J Neurol Sci* **437**, 120251 (2022).

63. Williams, D. T., LAfaver, K., Carson, A. & Fahn, S. Inpatient treatment for functional neurologic disorders. *Handbook of clinical neurology / edited by P.J. Vinken and G.W. Bruyn* **139**, 631–641 (2017).

64. LaFrance, W. C. *et al.* Treatment of psychogenic nonepileptic seizures (PNES) using video telehealth. *Epilepsia* **61**, 2572–2582 (2020).

65. Demartini, B. *et al.* A physical therapy programme for functional motor symptoms: A telemedicine pilot study. *Park. Relat. Disord.* **76**, 108–111 (2020).

66. Moseley, G. L. & Butler, D. S. Fifteen Years of Explaining Pain: The Past, Present, and Future. *J. Pain* **16**, 807–813 (2015).

67. Popkirov, S., Hoeritzauer, I., Colvin, L., Carson, A. J. & Stone, J. Complex regional pain syndrome and functional neurological disorders - time for reconciliation. *Journal of neurology, neurosurgery, and psychiatry* **90**, 608–614 (2019).

68. Kamper, S. J. *et al.* Multidisciplinary biopsychosocial rehabilitation for chronic low back pain: Cochrane systematic review and meta-analysis. *BMJ* **350**, h444 (2015).

69. Booth, J. *et al.* Exercise for chronic musculoskeletal pain: A biopsychosocial approach. *Musculoskelet. Care* **15**, 413–421 (2017).

70. Williams, A. C. de C., Fisher, E., Hearn, L. & Eccleston, C. Psychological therapies for the management of chronic pain (excluding headache) in adults. *Cochrane Database Syst. Rev.* **2021**, CD007407 (2020).

Resources

Online Resources

For health care professionals

FND Australia - fndaustralia.com.au

Digital Content

Health Care Professional Directory

Health Professional Resources (including Patient Workbook)

Patient Resources

Neurosymptoms - https://neurosymptoms.org/en/

Health Professional Resources

Patient Resources

FND standard of care
functional-neurological-disorder-standard-of-care.pdf (brighamandwomens.org)

Graded Motor Imagery

What is Graded Motor Imagery and How Can It Help Treat Chronic Pain? - Pathways

Graded Motor Imagery - Recognise App (Left Right Discrimination)

King's Health Partners Neurosciences Functional Neurological Disorder Masterclass – Occupational Therapy Role in Functional Neurological Disorders. Helen Jinadu. May 2019.

NOI group website and Explain pain resources: www.noigroup.com
Mindspot Pain Course: https://www.mindspot.org.au › courses › pain-course
ACI pain management network: aci.health.nsw.gov.au

Cognitive functional therapy: pain-ed.com

IASP and APS https://www.iasp-pain.org and www.apsoc.org.au
Persistent pain patient advocacy groups: Pain Australia and Australia Pain Management Association (APMA)

For patients

Department of Social Services offers advocacy regarding understanding or access the NDIS, Centrelink and other government services: National Disability Advocacy Program

Functional seizure management plan: https://fndaus.org.au/functional-seizure-management-plan/
For people with functional seizures, a seizure management plan may be helpful so others know how to best support the person. This seizure management plan was prepared by Dr. Katherine Gill, FND Australia Support Services Inc. with input and feedback by FND specialist Neurologists, Professor Jon Stone and Dr Alex Lehn; and members of FND Australia Support Services Inc. who live with functional seizures.

Patient Support Groups

FND Australia Support Services Inc – fndaus.org.au

FND Australia Support Services provides online informal patient peer to peer support groups and annual peer support group members workshops/webinars/focus groups provided by Master's students - Counselling/Psychotherapy and OT, the 12-week FND education and wellbeing "Kokoro Mollitia" program, a maintained listing of Australia wide health professionals/providers specialising in FND, training for MDT health professionals with little to no training delivered via video conferencing, in-service education workshop for health professionals. For FND patients and their carers who are in severe financial hardship they offer the low fee Counselling Program - yearly intake starts in February 2024. For NDIS applications that meet the NDIS criteria, they offer the low fee OT Program - intakes June and September 2024. The self-referral form for these programs is available on the website. All services and programs are delivered via Zoom video conferencing Australia wide. FND Awareness Day is the 25th of March held annually in Australia. Further information on programs or services are available on the website or email: info@fndaus.org.au.

FND Hope – fndhope.org

Useful Apps for patients

FND Aus App

The FND Aus App provides information on FND, including frequently asked questions. The FND Aus App provides information on FND, including frequently asked questions. It helps people to develop skills to manage their symptoms and improve wellbeing. The App offers a tracking function so people can track their physical and mental wellbeing, activity levels and goal achievements, alongside any changes in symptoms. The App includes a Functional Symptom Management Plan to assist bystanders, emergency professionals and carers, to support a person with an acute onset of a symptom e.g. functional seizure. Outputs can be shared with the person's treating team and used to identify potential symptom triggers. The app is free to download from the App store and Google Play store.

Neurosymptoms FND Guide

The Neurosymptoms FND Guide is a patients’ guide to Functional Neurological Disorder

Lehn A, *et al. BMJ Neurol Open* 2025; 7:e000970. doi: 10.1136/bmjno-2024-000970

(FND). It is an app version of neurosymptoms.org a website made by Professor Jon Stone and colleagues from the University of Edinburgh (Centre for Clinical Brain Sciences), a leading FND research centre.

Calm app and headspace app

The Calm and Headspace apps help with mindfulness, sleep, meditation and relaxation.

Smiling Mind app

A free app *for introduction to Mindfulness and Meditation. Mindfulness meditation helps to manage stress, improve resilience, general health and wellbeing.*

Professional Memberships

State-wide Rehabilitation Clinical Network: Functional Neurological Disorder Special Interest Group FND.SIG@health.qld.gov.au

Functional Neurological Disorder Society https://www.fndsociety.org/
